# The satellite observed glacier mass changes over the Upper Indus Basin during 2000–2012

**DOI:** 10.1038/s41598-020-71281-7

**Published:** 2020-08-31

**Authors:** Tariq Abdullah, Shakil Ahmad Romshoo, Irfan Rashid

**Affiliations:** Geoinformatics Department, University of Kashmir, Hazratbal, Srinagar, Jammu and Kashmir 190006 India

**Keywords:** Cryospheric science, Climate sciences, Environmental sciences, Environmental social sciences, Hydrology, Solid Earth sciences

## Abstract

Decadal glacier thickness changes over the Upper Indus Basin in the Jammu and Kashmir Himalaya were estimated using the TanDEM-X and SRTM-C Digital Elevation Models (DEMs) from 2000 to 2012. In the study area 12,243 glaciers having 19,727 ± 1,054 km^2^ area have thinned on an average of − 0.35 ± 0.33 m a^−1^ during the observation period. The highest thinning of − 1.69 ± 0.60 m a^−1^ was observed in the Pir Panjal while as the marginal thinning of − 0.11 ± 0.32 m a^−1^ was observed for the glaciers in the Karakoram. The observed glacier thickness changes indicated a strong influence of the topographic parameters. Higher thickness reduction was observed on the glaciers situated at lower altitudes (− 1.40 ± 0.53 m a^−1^) and with shallower slopes (− 1.52 ± 0.40 m a^−1^). Significantly higher negative thickness changes were observed from the glaciers situated on the southern slopes (− 0.55 ± 0.37 m a^−1^). The thickness loss was higher on the debris-covered glaciers (− 0.50 ± 0.38 m a^−1^) than on the clean glaciers (− 0.32 ± 0.33 m a^−1^). The cumulative glacier mass loss of − 70.32 ± 66.69 Gt was observed during the observation period, which, if continued, would significantly affect the sustainability of water resources in the basin.

## Introduction

The recent reports on the enhanced glacier-melt in the Himalayan region^1^ encompassing the Upper Indus Basin (UIB) and the consequent impacts on the water, food and energy security^2^ stimulated various scientific studies to investigate the health and dynamics of glaciers in the UIB^3–5,7–16^. However, most of the studies have focussed on the changes in the glacier area, snout recession, Equilibrium Line Altitude (ELA), impacts of the increasing temperatures, the influence of debris-cover^4,5^. Glacier mass balance is the most important indicator of glacier health and is regarded as the direct and immediate glacier response to any changes in climate^6^. However, glaciological mass balance, though considered more accurate method for glacier mass balance, is restricted to a few glaciers in the UIB owing to its rugged terrain, logistic challenges and security impediments^3^. In view of these constraints, the geodetic mass balance has emerged as a credible alternate approach to assess glacier mass balance changes at local and regional scales in the UIB^7–15^. The past studies have reported significant variability in glacier mass changes over the High Mountain Asia (HMA) region. Gardelle et al*.*^15^ for instance, provided comprehensive regional mass balance estimates over the Hindu-Kush Karakoram Himalaya (HKH) region between 2000 and 2011, and highlighted the higher mass wastage in the western Himalaya, moderate mass loss in the eastern and central Himalaya, small mass losses in the Hindu-Kush and stability or even slight mass gain in the Karakoram and Pamir sub-regions. The variability in the mass balance of glaciers in various Hindu-Kush Himalayan regions has been largely attributed to the peculiar topographic and climatic setting of each of the mountain ranges^16^.

However, it has been observed that the response of glaciers to climate change is quite variable, even within the same topographic and climatic regime^17^, complicating any generalization of glacier behaviour at local or regional scale^18^. The variable glacier response is partly explained by the micro-climatic niche of the individual glaciers in which they are set. However, when considered in tandem, the morphological and topographical characteristics like glacier elevation, slope, aspect, glacier size and supra-glacial debris cover largely explain the variable glacier response^19–21^. The morphological setting can enhance or delay the glacier response to climate variability^22^ which often complicates the understanding of glacier behaviour. Several studies have previously demonstrated the influence of morphological and topographical variables on glacier mass changes^23,24^. Copland et al*.*^25^ and Hewitt^26^ explained the variability of glacier mass balance as a function of various glacier morphological characteristics. A few researchers have also investigated the role of glacier morphological and topographic variables on glacier thickness changes, and demonstrated that the morphological and topographic variables can explain up to 35%^27^ and 25%^24^ of the glacier thickness changes. Salerno et al*.*^21^ on the other hand, indicated that the slope of a glacier tongue is the main topographic parameter controlling the glacier thickness changes. The influence of debris cover on the Himalayan glaciers is not very clear. Several geodetic mass balance studies have reported similar thinning rates for both debris-free and debris-covered glaciers^28^ contradicting the reports of the slowdown of glacier melting under thick debris-cover^29^.

**Figure 1:**
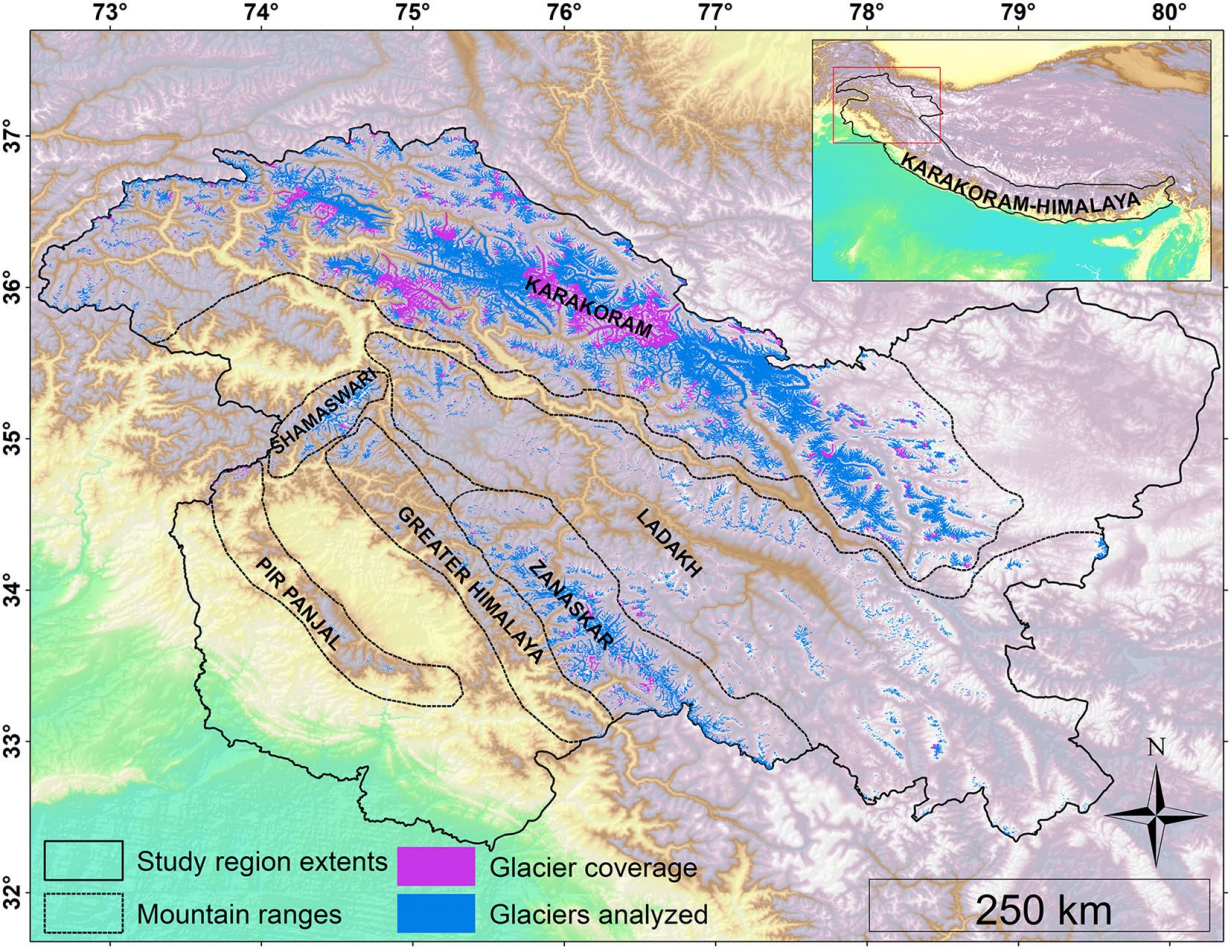
Location and topographic map of the study region. The distribution of RGI v 6.0 glacier cover analysed in the present study is represented by blue coloured polygons overlain on GTOPO30 DEM. The violet colour polygons represent total glacier coverage in the study region. Dashed-line polygons (modified after Shekhar et al*.*^63^) demarcate the mountain ranges. The black polygon demarcates the boundary of the study region. The Karakoram-Himalaya boundary is modified after Bolch et al*.*^3^. The figure was generated in ArcGIS version 10.4.1 (https://www.esri.com/en-us/arcgis/products/arcgis-pro/).

The present study investigated the changes in glacier thickness based on two publicly available SAR-based Digital Elevation Models (DEMs) from 2000 to 2012 over the UIB in the Jammu and Kashmir Himalaya, India (Fig. 1). The study area, comprising of Jammu, Kashmir and Ladakh regions, is spread over an area of 222,236 km^2^ and nearly 11% of the geographical area is covered by glaciers (glacier count = 15,064 spread over 24,022 km^2^)^30^. Majority of the glaciers in the UIB are valley glaciers except in the Ladakh mountain range where cirque glaciers are more dominant^31^. The study area is often divided in six mountain ranges: Pir Panjal range, (PPR), Greater Himalaya range (GHR), Shamaswari range (SR), Zanaskar range (ZR), Ladakh range (LR) and the Karakoram range (KKR) each with distinct climatic and topographic characteristics. The climatology of the region is largely dominated by the western disturbances (WDs) compared to the monsoons which are predominant in the Indo-Gangetic plains^32^. The WDs, mostly originating from the Mediterranean Sea, are the major sources of precipitation particularly during winters in the study region^33^. Glaciers in the western Himalaya encompassing the study region receive 60–70% of their annual accumulation from the WDs^34^.

### Data set

The Shuttle Radar Topography Mission (SRTM) operated between 11 and 22, February, 2000 and acquired Interferometric Synthetic Aperture Radar (InSAR) data from the space in two frequencies (X-band and C-band). However, compared to the C-band, the X-band system has limited coverage^35^. The 1-arc sec SRTM/X-SAR (X-SRTM) DEM is available from the German Aerospace Centre (DLR) and the non-void filled 3-arc sec SRTM/SIR-C DEM is distributed by the United States Geological Survey (USGS).

The TanDEM-X mission (TerraSAR-X-Add-on for Digital Elevation Measurements) with its twin satellites was launched in 2010 by the DLR. TanDEM-X and TerraSAR-X, flying in helix formation, record the backscattered signal with the satellite overpass lag as small as 3 s. The very small temporal baseline makes the data suitable for interferometric processing^36^. We used the TanDEM-X DEM composite data from 2010 to 2015 period and is referred to as 2012. The TanDEM-X 90 m DEM, released in September 2018, is a product variant of the global Digital Elevation Model (DEM) with the vertical accuracy up to 2 m^12^.

The glacier outlines, used in this study, are based on the globally complete Randolph Glacier Inventory (RGI) version 6.0^30^. Furthermore, the Supra-glacial Debris Cover Dataset v1.0, a global supraglacial debris-cover dataset was used for glacier debris-cover (DC) characterization^37^. Keeping in view the limitations of the debris-cover dataset, we manually corrected the discrepancies in the data for glaciers with debris-cover fraction > 19% using Landsat data. We also used the MODIS LST (MOD11A2) to characterize the climatic settings of different mountain ranges in the study region. The details of the dataset used in this study are given in Supplementary Table S2.

## Methodology

### DEM corrections and elevation changes

In order to remove the horizontal and vertical offset between the two DEMs, the universal co-registration algorithm was used^38^ (details in the Supplementary Section 1). Prior to their use for thickness change estimation, the DEMs were also corrected for radar penetration bias. The co-registered DEMs were differenced to generate the elevation difference (dH/dT) map over the glaciated terrain at pixel level. RGI6.0 glacier outlines^30^ were used to calculate the mean glacier elevation changes between 2000 and 2012 and the volume changes thereof. Using the density conversion factor of 850 kg m^−3^, the volume changes were then converted into glacier mass changes^39^ (details in the Supplementary Section 2). It is pertinent to mention that only the glaciers with dH/dT coverage > 30% were considered in the present study. Furthermore, the uncertainties in the glacier mass changes owing to the uncertainty in DEM differencing, radar signal penetration, uncertainty due to void fill, glacier outlines and mass conversion have been addressed separately and discussed in the Supplementary Section 3. The glacier thickness change estimates are based on the elevation differences in the DEMs obtained ~ 12 year apart. Though, the SRTM DEM was obtained over a shorter period of time (11–20 February, 2000) but the timestamp of each TANDEM-X acquisition is not same and is spread over a wider period. This has the potential to add to the uncertainty of glacier thickness and mass changes which has been considered in the uncertainty analysis in the present study^40^. The bias correction and uncertainty analysis is discussed in detail in the Supplementary Sections 3 and 4.

### Debris categorization and glacier topographical parameters

To assess the influence of supra-glacial debris cover, we used two criteria to differentiate between the clean and debris-covered glaciers: one proposed by Brun et al*.*^9^ using > 19% debris-cover fraction threshold (Criterion 1) for identifying debris and non-debris glaciers and the other proposed by Ali et al*.*^41^ which categorizes glaciers into three categories: clean glaciers with the debris-cover fraction < 25%; sparsely debris-covered glaciers with debris-cover fraction between ≥  25 and ≤ 50% and debris-covered glaciers with debris-cover fraction ≥ 50% (Criterion 2). Prior to the use for analysis, the debris-cover dataset was corrected manually for any discrepancy using Landsat satellite images, however, we only corrected the glaciers with debris-cover fraction > 19%. The topographic parameters like elevation, slope and aspect for each glacier in the study area were extracted from the TanDEX-X DEM in ArcGIS environment (details in the Supplementary Section 4).

## Results

### Glacier thickness and mass changes

**Figure 2:**
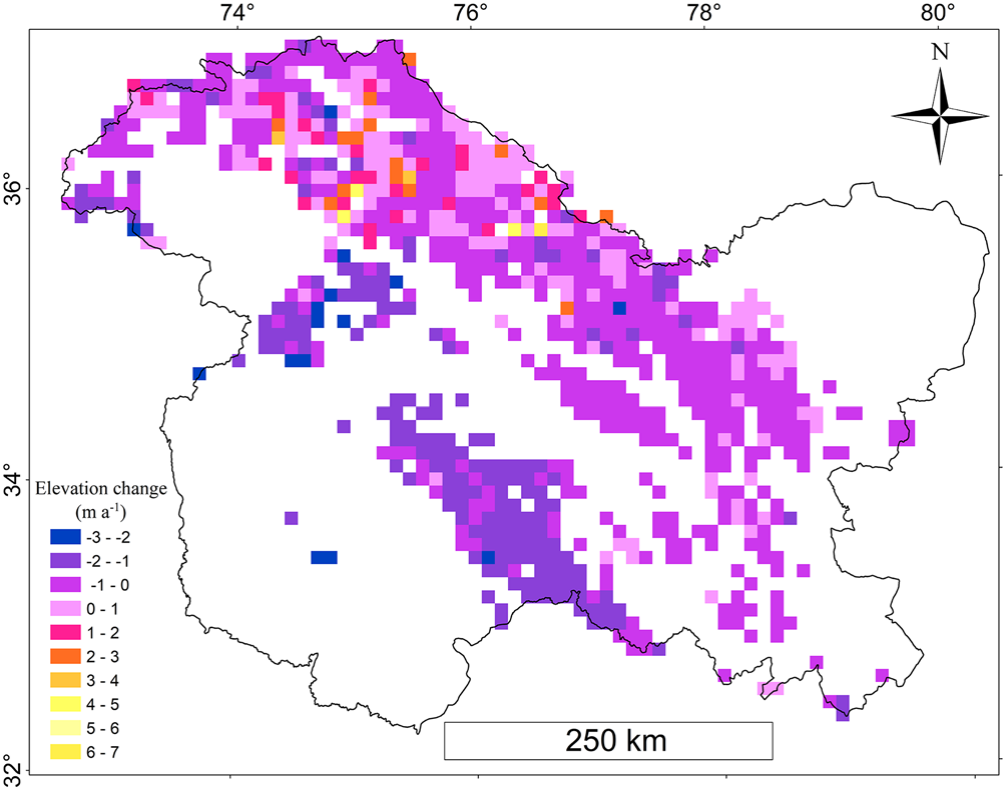
Gridded (10 × 10 km) glacier elevation changes over the study region. All the grid cells with fraction of glacier coverage < 2% were excluded from the map.

The investigation showed that the glaciers in the UIB were thinning at an average rate of − 0.35 ± 0.33 m a^−1^ (Table 1) amounting to the glacier mass loss of 297.5 ± 280.5 kg m^−2^ a^−1^. The mean glacier-wide thickness changes in the UIB varies from ~ − 5.0 to ~ 16.0 m a^−1^ (Fig. 2), however, it is pertinent to mention here that ~ 64% (count) of the glaciers, accounting for ~ 73.3% of the glacier area in the UIB, fall in the 0 to − 2.0 m a^−1^ thickness change category (Supplementary Fig. S3). The thickness change of − 0.11 ± 0.32 m a^−1^ is significantly lower in the KKR. The highest average glacier thickness loss of − 1.69 ± 0.60 m a^−1^ was observed in the PPR. The average glacier thickness loss of − 1.28 ± 0.46 m a^−1^ and − 1.12 ± 0.40 m a^−1^ was observed in the SR and GHR respectively. The glaciers in the ZR and LR have, on average, thinned − 1.17 ± 0.41 m a^−1^ and − 0.46 ± 0.26 m a^−1^ respectively (Table 1).

**Table 1:** Changes in glacier thickness across different mountain ranges of the study region.

Mountain (range)	Number ( N)	Fraction of dH/dT coverage (%)	dH/dT (m a^−1^)	Mass balance (m w.e. a^−1^)	Mass change (Gt a^−1^)	Mean elevation (m a.s.l)	Mean slope (°)	Debris cover (%)	Area (south aspect) (%)
KKR	5,579	78.52	− 0.11 ± 0.32	− 0.09 ± 0.27	− 1.32 ± 3.8	5,259	31.62	9.07	24.33
LR	3,717	92.93	− 0.46 ± 0.26	− 0.39 ± 0.24	− 0.96 ± 0.59	5,684	24.43	5.53	7.38
ZR	1,720	92.93	− 1.17 ± 0.41	− 0.99 ± 0.43	− 2.34 ± 0.84	5,032	23.59	13.14	26.41
SR	878	94.40	− 1.28 ± 0.46	− 1.08 ± 0.48	− 0.69 ± 0.26	4,724	22.27	14.77	19.19
GHR	243	94.10	− 1.12 ± 0.40	− 0.95 ± 0.42	− 0.88 ± 0.04	4,459	22.58	5.14	14.58
PPR	106	59.55	− 1.69 ± 0.60	− 1.43 ± 0.63	− 0.38 ± 0.01	4,153	20.68	8.69	8.53
UIB	12,243	82.12	− 0.35 ± 0.33	− 0.29 ± 0.29	− 5.86 ± 5.55	5,290	27.10	10.25	22.20

Only the glaciers with < 30% voids corresponding to an area of 19,727 km^2^ equivalent to ~ 82% of the total glacier area (24,022 km^2^) were considered for the analysis.

### Topographic influences

The investigation revealed a strong correlation between the mean glacier elevation and thickness change (R = 0.64). The influence of elevation on the glacier thickness change is evident from the fact that the highest glacier thickness loss (− 0.86 ± 0.56 m a^−1^) was observed in the glaciers located at mean elevations < 5,000 m a.s.l. and the rate of thickness loss is significantly lower (− 0.42 ± 0.33 m a^−1^) in the glaciers located at mean elevations > 5,000 m a.s.l. (Supplementary Fig. S6). It is pertinent to mention that ~ 25% and ~ 75% of the total glacier area is distributed at elevations < 5,000 m a.s.l. and > 5,000 m a.s.l. respectively.

The analysis also indicated a strong positive correlation (R = 0.87) between the mean glacier slope and thickness change, i.e., the thickness loss is less for glaciers situated on steeper slopes. The glaciers with the mean slope < 10° have thinned at the rate of − 1.52 ± 0.40 m a^−1^ while as, mass gain of 0.28 ± 0.40 m a^−1^ was observed in the glaciers with the mean slope > 30° (Supplementary Fig. S7).

40.64% of the glacier area under consideration in the UIB is distributed over the northern slopes and has thinned at the rate of − 0.22 ± 0.25 m a^−1^. The glaciers distributed on the eastern and western aspects (~ 37% of the glacier area) have thinned at the rate of − 0.30 ± 0.36 m a^−1^. The glaciers situated on the southern slopes, comprising ~ 22% of the UIB glacier area under consideration, have experienced the highest thickness loss (− 0.55 ± 0.46 m a^−1^), indicating a significant control of aspect on the glacier thickness changes (Supplementary Fig. S8). Analysis of topographic variables and their influence on glacier elevation changes across the six mountain ranges under consideration revealed a similar pattern as observed over the entire basin level (Supplementary Tables S3–S8). The detailed topographic and debris-cover characteristics of the glaciers in the study region are discussed in the Supplementary Section 5.

### Influence of supra-glacier debris and glacier size

The debris-covered glaciers showed more thickness losses compared to the clean glaciers in the UIB. Based on the criterion 2, ~ 95% of the glacier area in the UIB is under clean glaciers showing the ice thickness change of − 0.29 ± 0.33 m a^−1^. The sparsely debris-covered and debris-covered glaciers, as per the criterion, have receded more at the rate of − 0.78 ± 0.33 m a^−1^ and − 0.47 ± 0.41 m a^−1^ respectively (Supplementary Table S6). The significant impact of the debris-cover on the glacier thickness change is also evident when using the Criterion 1 for the debris-cover categorization. The clean and debris-covered glaciers based on the criterion 1, comprising ~ 92% and ~ 8% of the glacier area, have witnessed the thickness change of − 0.35 ± 0.33 m a^−1^ and − 0.53 ± 0.35 m a^−1^ respectively (Supplementary Table S6).

There is a positive, though weak, relationship (R = 0.25) between the glacier size and thickness changes. In general, the smaller glaciers experience higher thickness losses compared to the larger glaciers^42^. The larger glaciers with the size > 50 km^2^, comprising ~ 36% of the glaciated area under consideration in the UIB, have shown the lowest thickness changes (− 0.32 ± 0.26 m a^−1^). On the contrary, the glaciers with area < 50 km^2^ comprising ~ 64% of the total glacier area have thinned, on an average, − 0.43 ± 0.26 m a^−1^ during the observation period (Supplementary Table S7), indicating a significant impact of the glacier size on the glacier thickness changes.

### Glacier elevation changes and prevalent climatic conditions

Analysis of the MODIS LST temperature from 2000–2014 revealed distinct climatic conditions of the different mountain ranges in the study region. Relatively higher winter (Nov.–April) and summer (May–Oct.) temperatures of − 4.35 °C and 16.45 °C compared to rest of the mountain ranges were observed in the PPR range (Table S8). On the other hand, lowest winter (− 15.49 °C) and summer (1.35 °C) temperatures were observed over the KKR. The GHR exhibited − 4.5 °C and 14.52 °C temperature during winter and summer seasons respectively. With the mean annual temperature settling at 5.38 °C, the winter and summer temperature of − 6.1 °C and 16.32 °C were observed in the SR. Almost similar winter and summer temperatures were observed for the ZR and LR with − 11.12 °C and − 11.55 °C during winters and 12.72 °C and 12.83 °C during summer respectively (Supplementary Table S8). The relatively higher elevation changes observed over the PPR and the lowest elevation changes in KKR are in agreement with the prevalent climatic regimes in the two ranges (Table S8). Similarly, the elevation change pattern observed in the GHR, SR, ZR and LR is also in line with the prevalent climatic settings observed across these mountain ranges. The details of the average summer and winter temperatures observed over different mountain ranges in the study region from 2000 to 2014 are provided in the Supplementary Table S8.

### Validation of the results

Field observations of mass balance over the study region are very scarce. In fact only 4 glaciers over the study region have been studied for mass balances^43^. These observations are, however, very short (one year mass balance for the Kolahoi and Shishram, 2 years for the Rulung and 8 years for the Nehnar glacier respectively) and restricted to 1980s and as such were not considered for the validation in the present study. The elevation changes observed in the present study were, however, validated against the elevation changes based on the High Mountain Asia (HMA) DEM with specific date-stamps and SRTM DEM differencing^12^. The HMA-SRTM elevation changes for the selected glaciers fall within the uncertainty bars of the TanDEM-X-SRTM derived elevations changes observed in this study. The spatially distributed elevation changes of these glaciers along with the GLIMS ID are provided in Supplementary Fig. S10.

## Discussion

Topographical parameters, to a large extent, explain the thickness change variability observed across the six mountain ranges in the UIB. This is due to the fact that there is a marked difference in the topographical variables across the ranges which is strongly related to glacier response time and sensitivity of mass balance to climate change^18,21^. The glaciers situated on the gentle slopes have relatively longer response time, leading to the slow glacier dynamics^44^. They do react immediately to warming by retreating, however, they remain in equilibrium by making dynamic adjustments to climate forcing and therefore remain out of balance for a longer time^45^. The relationship between the glacier slope and the observed glacier thickness change is strong (R = 0.76).

The glacier aspect has a profound effect on the glacier melting^20,46^. In the Himalaya, the north facing slopes generally receive less solar radiations compared to the south facing slopes^47^. This explains the robust relationship (R = 0.86) observed between the aspect and the glacier thickness changes in all the six ranges of the basin. Relatively higher melting rates observed for the glaciers situated on the warmer southern slopes have been previously reported in the Himalaya^47^.

The glaciers situated at lower altitudes are more sensitive to the rising temperatures, as these glaciers tend to have larger mass turnover to balance the relatively higher melting in their lower reaches^48^. This explains the influence of glacier elevation on the observed ice thickness changes as evident from the high correlation coefficient (R = 0.78). Similar relationship has been previously reported in the Himalayan region^9,49^. Contrary to the common sense, the glaciers with mean elevation > 6000 m a.s.l. have shown higher ice thickness losses compared to the glaciers situated at lower altitudes between 5,000 and 6,000 m a.s.l.. This asymmetry is explained by the fact that nearly ~ 35% of the total glacier area above 6,000 m a.s.l. is south-oriented compared to ~ 23% of the south-oriented glaciers situated between 5,000 and 6,000 m a.s.l. elevation. The shallow mean slope of the glaciers situated between 4,000 and 5,000 m a.s.l. elevation explains the slightly higher observed ice thickness recession compared to that of the glaciers located at elevations below 4,000 m a.s.l. (Supplementary Fig. S6).

The higher ice thinning (− 1.69 ± 0.60 m a^−1^) observed in the PPR correlates with its lower mean altitude compared to the other mountain ranges in the UIB (Table 1). The relatively higher glacier thinning observed in the SR despite its higher mean elevation compared to that of the glaciers in the GHR is explained by the presence of the relatively more supra-glacial debris of the glaciers in the SR range (Table 1).

The variable glacier thickness changes observed in the climatologically somewhat similar LR and ZR ranges^50^ situated in the cold desert region of Ladakh are also attributed to the differential topographic and morphological settings of the glaciers in these two ranges. The glaciers in ZR have relatively lower mean altitudes (5,032 m a.s.l.), relatively more area distributed on the southern slopes (~ 26%) and relatively higher (~ 13%) debris-cover (Table 1). All these parameters favour enhanced glacier melting and therefore explain the relatively more thinning of the ZR glaciers compared to the glaciers in the LR. Almost zero glacier thickness changes observed in the KKR are in line with the previous studies in the mountain range^8,10,13–15^. The stability of the glaciers in the KKR, despite the fact that the morphological and topographical variables (Table 1) in general seem to favour enhanced melting, corroborate with the concept of the preponderance of climatic influence on the KKR glacier dynamics^51–53^.

The influence of the glacier morphological and topographic variables on glacier mass balance has been previously reported by Huss^27^, who concluded that 35% of the glacier mass balance variability is explained by the median glacier elevation, mean glacier tongue slope and glacier area. Similarly, Rabatel et al*.*^24^ reported that 25% of the glacier mass balance variability is explained by glacier median elevation and mean slope of the glacier tongue.

Among the non-climatic parameters, supra-glacier debris is considered as one of the important parameter influencing glacier mass balance^21,54^. Supra-glacial debris cover alters glacier surface energy balance acting as a barrier between the atmosphere and the ice. It can lead to the reduction of melt, but in case of thinly debris-covered glaciers or the glaciers with patchy supra-glacial debris, melt rates are enhanced compared to the bare ice^55^. Differential thinning of debris-covered and clean glaciers is reported in several other studies^26,56^, however, the picture is not very clear as the debris influence on the melting of glacier varies as a function of the debris-cover extent, distribution and thickness^42^. Some studies have suggested reduced melting of the debris-covered glaciers^56,57^, and contrarily, a few other researchers have reported enhanced melting from the debris-covered glaciers^58,59^. Even some studies have reported similar thinning rates from both debris-covered and debris-free glaciers indicating no overall influence of the debris-cover on mass balance^28^. The findings of this study are more or less in tune with the studies suggesting relatively higher thinning rates from the debris-covered glaciers which is corroborated by the higher mass balance sensitivity of the debris-covered glaciers to the rise in temperature^60^. However, the influence of debris-cover on the glacier thickness changes is not uniform; out of the 1,011 debris-covered glaciers (based on the criterion 1), 275 glaciers showed stability or even a slight gain in thickness, while as 736 glaciers showed the negative thickness changes. The observed variability in the glacier thickness changes from the debris-covered glaciers is explained by the variability of topographic variables; the glacier with positive or no-change in the ice thickness are situated at higher mean elevation (5,431 m a.s.l.) and have steeper slope (~ 29°) compared to the glaciers showing the negative thickness changes which have a mean elevation of 5,228 m a.s.l. and ~ 24° slope (Supplementary Fig. S9).

Larger glaciers are laggard in responding to climate perturbations because of their long response time, with the consequent lower retreat rates^42^. The results indicate a slight positive correlation between the glacier size and the glacier thickness changes, however, the relationship is not significant or even uniform across the different glacier size categories (Supplementary Table S7). The observed variation in the glacier thickness for different glacier-size categories is largely controlled by the topographic variables. For instance, the glaciers with area < 1 km^2^ have thinned relatively less due to their location at the higher mean elevations (5,292 m a.s.l.). The observed lower thinning rates of the smaller glaciers is corroborated by the fact that the smaller glaciers across the mountain ranges in the UIB are situated at higher altitudes, mostly in cirques and below the rock cliffs where the accumulation is significantly pushed up by snow avalanches and wind drift^61,62^, resulting in the slowdown of the glacier thinning. Generally, the lower mean elevation, shallower slopes, mean southern aspects and relatively higher debris-cover fraction of the glaciers (Supplementary Table S7) favour the glacier thinning^45,47,48^ which explains the observed higher thinning (− 0.58 ± 0.34 m a^−1^) of the glaciers in 40–50 km^2^ size-category compared to the average thinning of − 0.34 ± 0.22 m a^−1^ observed in the relatively smaller glaciers (30–40 km^2^). The results are in agreement with the numerous studies conducted in the Himalaya and elsewhere which have investigated the influence of glacier size on glacier recession^4,5^.

The differential elevation changes observed across different mountain ranges were also found in good agreement with the prevalent climatic regimes over the mountain ranges (Supplementary Table S8). It is pertinent to mention here that Shekhar et al.^63^ based on the analysis of 18 meteorological stations distributed over different mountain ranges of the study region reported an increase in both the maximum and minimum temperatures except for the KKR. The study reported an increase of 0.8, 2.0 and 1.0 °C in the maximum temperature in the PPR, SR and GHR respectively from 1988 to 2008. The minimum temperatures during the same period increased by 0.6, 1.0 and 3.4 °C in the PPR, SR and GHR mountain ranges respectively. However, the study reported a decrease of around 1.6 and 3.0 °C in the maximum and minimum temperatures respectively over the KKR. A decrease of ~ 280, 80 and 440 cm in the precipitation was also reported over the PPR, SR and GHR respectively during the same period^63^. The study also reported a marginal decrease of ~ 40 cm in the snowfall in the KKR during the period. The stability of the glaciers in the KKR is attributed to the decreasing temperatures and almost negligible decrease in the seasonal snow fall observed in the region.

The thickness change estimated in this study (− 0.35 ± 0.33 m a^−1^) is largely in agreement with the previous studies carried out in the region with slight variations^7–10,13,15^. The observed elevation change estimates are lower when compared to the estimates of − 0.6 ± 0.09 m a^−1^ (2003–2008) by Kääb et al.^65^ and − 0.50 ± 0.28 m a^−1^ (2000–2012) by Vijay and Braun^13^. However, the use of sparse ICESat altimetry and SRTM DEM data and the different observation period (2003–2008) by Kääb et al.^65^, unlike the DEMs of same resolution used in this study, explains the deviation of the two ice-thickness change estimates. The observed deviation may also be due to the different DEM resolutions and processing techniques used in the two studies. In the case of Vijay and Braun^13^, though the data and the period of observation are same, but the ice-thickness estimates are based on a small subset (2,308) of the large number of glaciers used in this study (12,243). The glacier thickness changes estimated in this study agree well with the previous studies showing stability or even mass gain in the Karakoram and negative changes in the rest of the Himalaya^8,9,13,15^. The observed ice thickness changes of − 0.11 ± 0.32 m a^−1^ over the KKR agree reasonably well with the estimates of + 0.12 ± 0.19 m a^−1^ (2000–2008, 2000–2010) reported by Gardelle et al*.*^15^; − 0.03 ± 0.04 m a^−1^ by Kääb et al*.*^65^; and − 0.19 ± 0.22 m a^−1^ by Vijay and Braun^17^. The results are however relatively higher, though with significant intra basin variations, when compared with the average glacier thickness change estimate of − 0.21 ± 0.05 m a^−1^ for the Hindu Kush-Karakoram-Himalaya (HKKH)^65,66^.

## Conclusion

In this study, the glacier thickness changes over the UIB in the Jammu and Kashmir Himalaya were quantified using the SRTM-C and TanDEM-X Digital Elevation Models (DEMs) during the period from 2000 to 2012. The study concluded that the glaciers in the region have thinned at the rate of − 0.35 ± 0.33 m a^−1^ which amounts to the glacier stored water loss of 70.32 ± 66.69 Gt during 12 year observation period. The variability in the observed glacier thickness changes across the six mountain ranges in the region is explained well by the variability of topographic parameters across the ranges. However, further investigations aimed at understanding the range-wise glacier response to climatology would provide further insights into the differing glacier behaviour and response observed over the topographically complex mountainous UIB. The glacier thickness changes across different mountain ranges of the data scarce Jammu and Kashmir Himalaya presented in this study is vital for determining the sustainability of water resources in the UIB.

## Supplementary information

Supplementary Information.

## Data Availability

The dataset will be available from the corresponding upon request.
