# Supplementary material for: The satellite observed glacier mass changes over the Upper Indus Basin during 2000–2012
Source: Sci Rep. 2020 Aug 31;10:14285. doi: 10.1038/s41598-020-71281-7 (PMC7459122; doi:10.1038/s41598-020-71281-7)
Supplement: Supplementary file 1 — Supplementary Information. [file 41598_2020_71281_MOESM1_ESM.pdf]

SUPPLEMENTARY MATERIAL

The Satellite Observed Glacier Mass Changes over the Upper Indus Basin during 2000-2012

Tariq Abdullah, Shakil Ahmad Romshoo* and Irfan Rashid

Geoinformatics Department, University of Kashmir, Hazratbal Srinagar, Jammu and Kashmir, India – 190006

*email: shakilrom@kashmiruniversity.ac.in

1. DEM CO-REGISTRATION

Even though acquired over the same terrain, there is some offset in the horizontal and vertical direction of the two DEMs (Tandem-X and SRTM-C DEM) and therefore, the two DEMs are co-registered before using them for any analysis. The subpixel offsets, affecting the DEM comparison[1], were corrected employing the universal co-registration algorithm[2]. The algorithm uses a slope normalized cosine relationship between aspect and elevation change (DEM difference) to minimize the offsets as follows:

$$\frac{dH}{\tan(\alpha)} = a.\cos(b - \Psi) + C \quad (1)$$

$$C = \frac{\overline{dH}}{\overline{\alpha}} \quad (2)$$

where α is slope; Ψ is glacier aspect; and the variables a, b, and c are the magnitude, direction, and mean bias respectively. dH and $\overline{dH}$ is elevation difference and overall elevation bias respectively. The minimization process was repeated till either the magnitude of shift (a) was <0.5m or the normalized median absolute difference (NMAD) on off-glacier terrain was <5% than the previous pass[2]. The whole offset minimization process was executed using the python implementation of the co-registration algorithm for TanDEM-X and SRTM-C[3]. For co-registration, the study region was divided into two parts; one roughly covering the KKR and LR mountain ranges and the other covering the rest of the study region.

2. DEM differencing, void filling and bias correction

The co-registered DEMs were differenced to generate the elevation difference (dH/dT) map over the glaciated terrain at pixel level. There is no consensus on glacier-wide void threshold to exclude a glacier for elevation change analysis. For example, some researchers have excluded glaciers with voids >20%[4], while as the others have used a void threshold of 30%[5, 6]. In the present study, we applied a void threshold of 30% to exclude the glaciers from the analysis. This reduced the glacier dataset from 15064 (based on the RGI v6.0 glacier inventory) to 12243. The voids were filled after the DEM differencing instead of using the original DEM[7]. Again, there is no standard interpolation technique recommended to fill the voids[8]. In this study, we produced up to 35% false voids for a set of six glaciers[8] and filled them using two interpolation methods; Natural Neighbour (NN)[9] and Inverse Distance Weighting (IDW)[10] in the GIS environment. Both the algorithms produced reasonable results with a deviation of 0.07 m (for NN) and 0.10 m (for IDW) from the mean value

for the glaciers prior to the void filling. Accordingly, NN algorithm was used to fill the voids. RGI v6.0 glacier outlines[11] were used to calculate mean glacier elevation changes from the difference between 2000 and 2012. The thickness changes were also calculated for each 10x10 km grid cell for visualisation over the entire study area. It is pertinent to mention that the elevation changes are often associated with the sensor and terrain specific biases inherent to the DEMs[12]. The errors in the SRTM-C and TanDEM-X are largely related to the terrain characteristics[13]. However, various studies[14, 15] have reported a very few sensor and terrain specific biases in the TanDEM-X data and therefore, it is assumed that the biases in elevation difference map are largely due to the errors in the SRTM-C DEM[16-18]. Various studies have suggested correction of the terrain related biases using polynomial and regression fitting[19, 20]. These studies did highlight the pronounced biases with increasing elevation and slope. The elevation difference between X and C SRTM ($SRTM_{C\text{-band}}$ - $SRTM_{X\text{-band}}$) is often used as first order approximation of radar penetration[21]. Though, the penetration bias varies from region to region, however, in several studies when the appropriate SRTM X data was not available, the penetration bias estimates reported for the neighbouring regions have been adopted[22]. In view of the fact that sufficient SRTM-X band is not available for the study region, we therefore calculated the signal penetration bias after a recent study by Vijay and Braun[23] carried out over the Lahaul-Sipti region of western Himalaya. The penetration bias was calculated using the following exponential function:

$$y = 7E - 06e^{0.0023X} \qquad (3)$$

where ‘x’ is absolute surface elevation and ‘y’ is the relative penetration bias between SRTM X and C band.

Since TanDEM-X DEM is a stacked product with the data acquired over multiple seasons and years as such a uniform radar penetration is unlikely, further owing to the higher frequency of X band, therefore less penetration, the bias and related uncertainty of a potential radar penetration was ignored in the present study[24]. We also found very negligible mean off-glacier elevation difference of 0.06 m a^{-1}, highest (~0.30 m a^{-1}) in the GHR, SR and PPR ranges and ~0.15 m a^{-1} for the LR and ZR ranges and the least of ~0.05 m a^{-1} for the KKR. The off-glacier glacier elevation changes and their dependence on slope are presented in Fig. S11 and Fig. S12. The off-glacier elevation bias together with the radar penetration corrections calculated as a function of altitude (Equation 3) were applied on the SRTM-TanDEM-X elevation differences. We used the average

ice density of 850 kg m^{-3} to convert the ice-thickness changes to mass changes which has been widely used for glacier mass budgeting over Himalayas and elsewhere[25].

3. Accuracy assessment

We followed the methodology for uncertainty assessment after Huber *et al.*[24] with additional term to account for the radar penetration error ($\sigma_{penetration}$) assuming that all the errors are uncorrelated and random. The uncertainty of glacier-wide specific elevation change (Δ_h) is computed as:

$$\delta_{\Delta h} = \sqrt{\sigma_z{}^2 + \sigma_{voidfill}{}^2 + \sigma_{TDXdate}{}^2 + \sigma_{penatration}{}^2} \quad (4)$$

The σ_z, $\sigma_{TDXdate}$ and $\sigma_{penatration}$ are uncertainty of elevation change rates, temporal uncertainty of TanDEM-X and uncertainty of radar signal penetration respectively.

In addition to the glacier elevation change uncertainty ($\delta_{\Delta h}$), the uncertainty of glacier area (δ_A) and the error in the density assumption (δ_ρ), are also taken into consideration for estimating the uncertainty of the mass change ($\delta_{\Delta M}$), which is calculated as follows:

$$\delta_{\Delta M} = |\Delta_M|.\sqrt{\left(\frac{\delta_{\Delta h}}{\Delta h}\right)^2 + \left(\frac{\delta_A}{A}\right)^2 + \left(\frac{\delta_\rho}{\rho}\right)^2} \quad (5)$$

The uncertainties in in elevation change rates are estimated by evaluating the off-glacier elevation changes[26]. In the present study the elevation changes were corrected for outliers by widely used Normalized Median Absolute Deviation (NMAD) approach[27] and subsequently used in several glaciological studies[22, 23, 25].

$$NMAD = 1.4826.median.[|\Delta hj - m\Delta h|] \quad (6)$$

Where, Δ_{hj} and $m\Delta_h$ denotes the individual elevation changes and median of all the Δ_{hj} respectively. The influence of the outliers is significantly minimised in the NMAD approach and hence is a preferred statistical uncertainty estimator[27]. The NMAD derived at 5° slope bins on off-glacier area (Fig. S12) exhibited strong dependence on slope, however, we did not restricted our error analysis to a slope threshold[23]. This off-glacier NMAD was used to calculate NMAD for the glaciated area and each glacier. The NMAD was calculated using the Zonal statistics function in the ArcGIS environment considering off-glacier area ranging between 300-1500 m around each glacier. Since the zonal statistics function, the option for calculation of some statistical parameters including median (required for calculation of NMAD) is not available for floating point values, each elevation change pixel (90 m x 90 m) was therefore scaled by a factor of 10 and stored as an integer

data set. Once the calculations were done, the resulting NMAD values were rescaled back to original values. The final uncertainty in elevation change rates (σ_z) was calculated using the widely accepted approach[5, 19, 20, 26], considering the spatial autocorrelation as:

$$\sigma_z = \begin{cases} \sigma \Delta h \sqrt{\frac{A_{Cor}}{5A}} & , A \geq A_{Cor} \\ \sigma \Delta h, A < A_{Cor} \end{cases} \quad (7)$$

where $\sigma\Delta h$ is the off-glacier NMAD, A is the glacier area analysed in the study area and Acor = πd^2, with d being the decorrelation length. We analysed the off-glacier elevation changes in the study region and the spatial auto-correlation of the off-glacier elevation changes was calculated using the Spatial Autocorrelation tool (Moran's I based correlogram analysis) in the GIS environment[28]. We determined mean value d=950 for the study region. The Moran's I value of ~0.54 indicates that the spatial autocorrelation is not very strong though. For the spatial autocorrelation the study region was divided roughly into 4 parts, part 1 comprising the KKR region, part 2 comprises the LR and ZR regions. Part 3 include the SR and GHR mountain ranges and the part 4 is based on the PPR mountain range. The spatial autocorrelation was computed on 90 m grinds for 20,000 m scales separately for each of the four parts. The uncertainty introduced due to the void filling was assumed to be equal to the difference of ± 0.07 m in the mean value of six glaciers before and after filling the false voids. The uncertainty due to the TanDEM-X date ($\sigma_{TDXdate}$) was assumed to be equal to be ± 2 times the annual elevation change rate from 2000 to 2012[24]. Further, we assumed the uncertainty in radar penetration as high as the correction factor itself after Huber *at al.*[24]. The uncertainty of the glacier area was estimated using the approach proposed by Braun *et al.*[29] and is described by the following equation.

$$\delta A = \frac{R_P/_A}{R_P/_{A_{Paul\ et\ al. 2013}}} \quad (8)$$

Where δA is glacier area uncertainty, $R_P/_A$ is the glacier perimeter-area ratio and $R_P/_{A_{Paul\ et\ al.2013}}$ is a constant equal to 5.03 km^{-1} [30].

Uncertainty error of 5.3% was estimated for the glacier area. It is pertinent to mention here that though the RGI v6.0 glacier outlines were corrected for any noticeable discrepancies, however, we almost doubled the error estimates (10%)[31] to account for the temporal evolution of the glacier

extents during the period of investigation[32] and the errors due to the offsets between the timestamps of the source images used for RGI glacier delineation (1998-2009 in our case) and the DEM timestamps[33]. A constant value of ± 60 kg m^{-3} was used to account for the uncertainty associated with the volume to mass conversion[34]. The cumulative errors due to all the three factors were taken into account for the glacier mass change estimates. Furthermore, to assign the uncertainties for a sample average (for example glaciers in the elevation range in a mountain range), we calculated the uncertainty of the sample-wide elevation change ($\delta_{\Delta hsample}$) using the following formula[24]:

$$\delta_{\Delta hsample} = \frac{\sum \sigma \Delta h}{n} \qquad (9)$$

where Δh is the mean uncertainty of each item i (pixel) and n is the number of items (pixels) in the sample.

4. Debris categorization and glacier topographical parameters

There is no consensus regarding the criteria for the designation of a glacier as a debris-covered or a clean glacier[25, 35]. Several studies[36, 37] have considered glaciers with fully or partially debris-covered ablation zones as debris-covered glaciers. Xiang *et al*.[38] and Janke *et al.*[39], on the other hand, used thresholds of 5% and 25% debris-cover area fraction to define the debris-covered glaciers. Brun *et al.*[25] used the debris-cover fraction threshold of 19% for debris-covered glacier definition. In view of the variable thresholds, the present study therefore used two criteria to differentiate between the clean and debris-covered glaciers: one proposed by Brun *et al.*[25] using >19% threshold (Criterion 1) for debris-cover glacier definition and the other proposed by Ali *et al.*[35] which categorizes glaciers into three categories: clean glacier with debris-cover fraction <25%; sparsely debris-covered glaciers with debris-cover fraction between ≥25% and ≤50% and debris-covered glaciers with debris area ≥50% (Criterion 2). The glacier topographic parameters like elevation, slope and aspect for each glacier in the study area were extracted from the TanDEM-X DEM in ArcGIS environment. The topographic parameters, glacier morphological characteristics (glacier size) and debris-cover were used to assess their influences on the observed glacier thickness and mass changes in the basin.

5. Topographic variables and supra-glacier debris characteristics

In view of the heterogeneous topography, the study area was divided into six known mountain ranges[40], each having a unique geomorphic and climatic setting, and the ranges offer specific niches for the existence of glaciers in the region. As per the inventory data used in this study, the glaciers numbering 15064 (as on year 2000 ± 2) in the UIB, cover an area of 240221401 km^2

(~11% of the geographical area), the highest in the Indian Himalayas. The recent inventories still contain significant differences in the number and area of glaciers reported from the basin[41-45]. These differences arise from different methodological approaches, difficulties such as the presence of snow- and cloud-cover on available satellite scenes, the treatment of debris-covered glaciers, and, in particular, the different definitions of glaciers[30, 46]. The glaciers in the UIB, investigated in the present study, range in size from 0.01 km^2 to 1077.95 km^2, with an average size of 1.6 km^2. These glaciers in the basin are spread over a wide altitudinal range between 3200 m and 7300 m a.s.l., however, most of the glaciers 90% (count) are concentrated in the elevation range of 4500-6000 m a.s.l..

6. Glacier hypsography

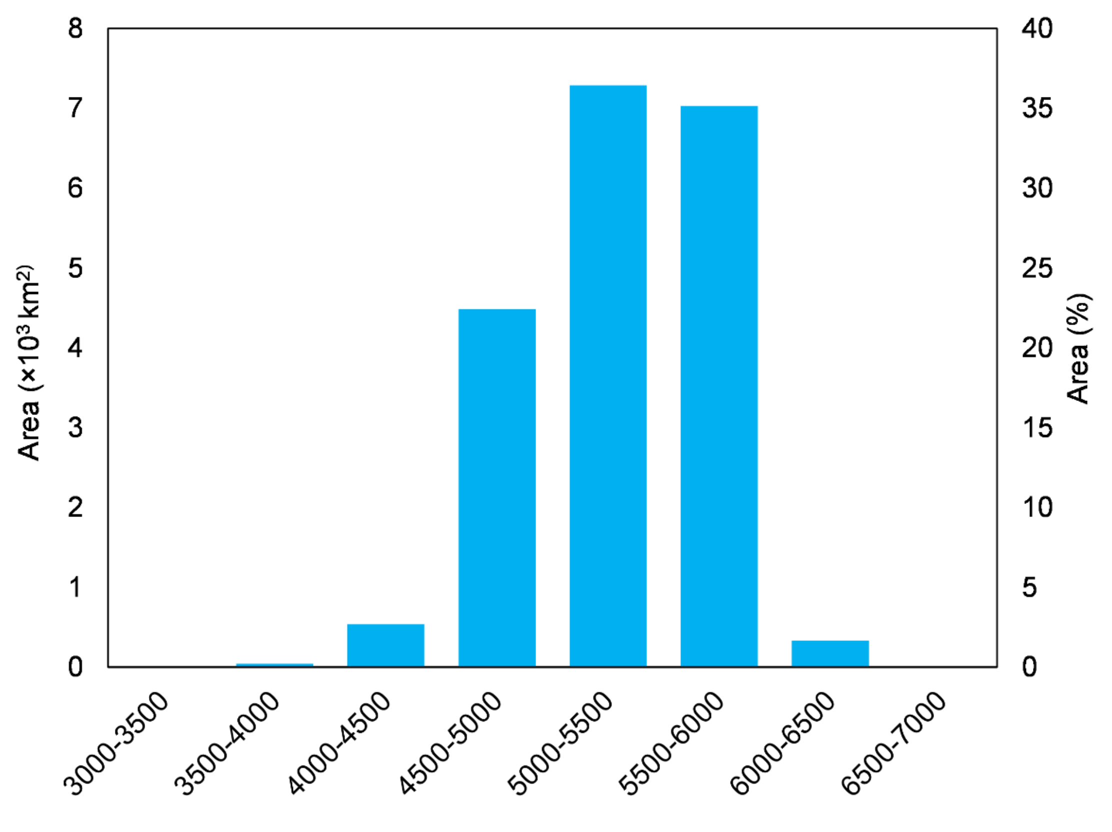

Supplementary **Fig. S1:** Distribution of glacier cover in different elevation ranges. In the figure the glaciers are grouped together in the elevation bins based on the mean elevation of individual glaciers.

The elevation ranges of 4500-5000 m, 5000-5500 m and 5500-6000 m a.s.l. harbour ~23%, ~37% and ~35% of the glacier cover respectively. The glaciers with mean elevation <4500 m a.s.l. and

>6000 m a.s.l. comprise only ~3% and ~2% of the total glacier area respectively. The mean glacier slope in the UIB varies between 7° and 63°. The analysis of the slope further revealed that nearly 62% of the glacier cover has mean slope between 20° and 30° (Fig. S2).

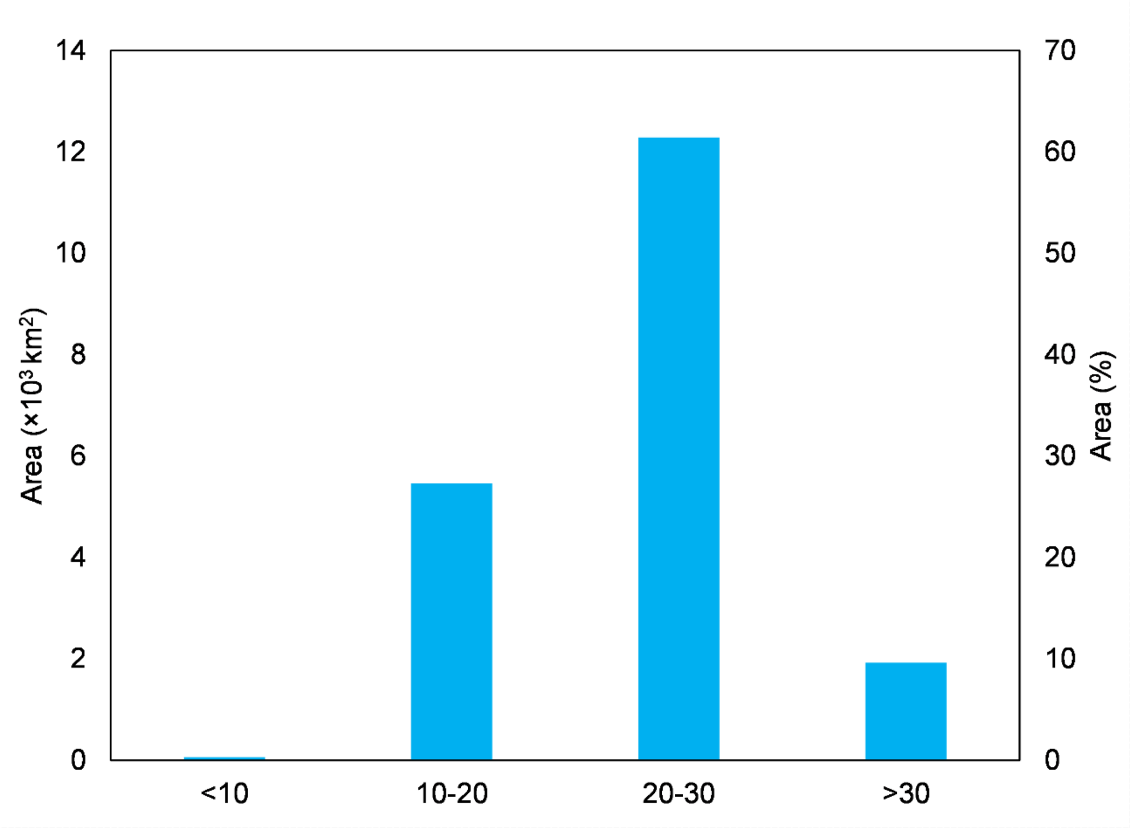

Supplementary **Fig. S2:** Glacier distribution over different slopes. In the figure, the glaciers are grouped together in the slope bins based on the mean slope of individual glaciers.

Around 27% and 10% of the total glacier area is situated on the mountain ranges with slopes between 10° to 20° and >30° respectively. Around 40% of the glacier cover have mean norther aspect, ~22% have mean southern aspects, ~16% and ~20% of the glacier area is distributed on western and eastern aspects respectively. The analyses revealed that ~14% of the total glacier area in the basin is covered with varying extents of supra-glacier debris. Using a threshold criterion of 19% debris-cover fraction to distinguish between the clean and debris-covered glaciers, the analysis showed that the debris-covered glaciers occupy ~13% of the total glacier area in the basin. Contrarily, the categorization based on the threshold of 25% debris-cover fraction indicates that the debris-covered glaciers comprise only 5.5% of the total glacier area. This study also highlighted the control of topographical variables on the debris-cover extent[47]. The supra-glacier

debris decreases with the increasing glacier altitude and the relationship is particularly significant (R=-0.79) at elevations >4500 (Table S1). We also found a negative but weak correlation, between the debris-cover and mean glacier slope. The investigation revealed that the debris-covered glaciers are generally situated at the lower altitudes (5253 m a.s.l.) in the basin and have relatively shallower mean slope (25°) compared to the clean glaciers with the mean altitude of 5300 m a.s.l. and mean slope of 28° (based on the Criterion 1). Relationship of debris-cover with other parameters like glacier aspect and glacier area could not be established.

Supplementary **Table S1:** Distribution of glacier debris on the basis mean glacier elevation.

Elevation category (m a.s.l.)	Debris cover (%)
<3500	0.0267
3500-4000	0.915145
4000-4500	6.636534
4500-5000	35.79417
5000-5500	38.16361
5500-6000	17.58546
6000-6500	0.92106
>6500	0

7. **Glacier thickness changes in the study region** (Supplementary Fig. S3)

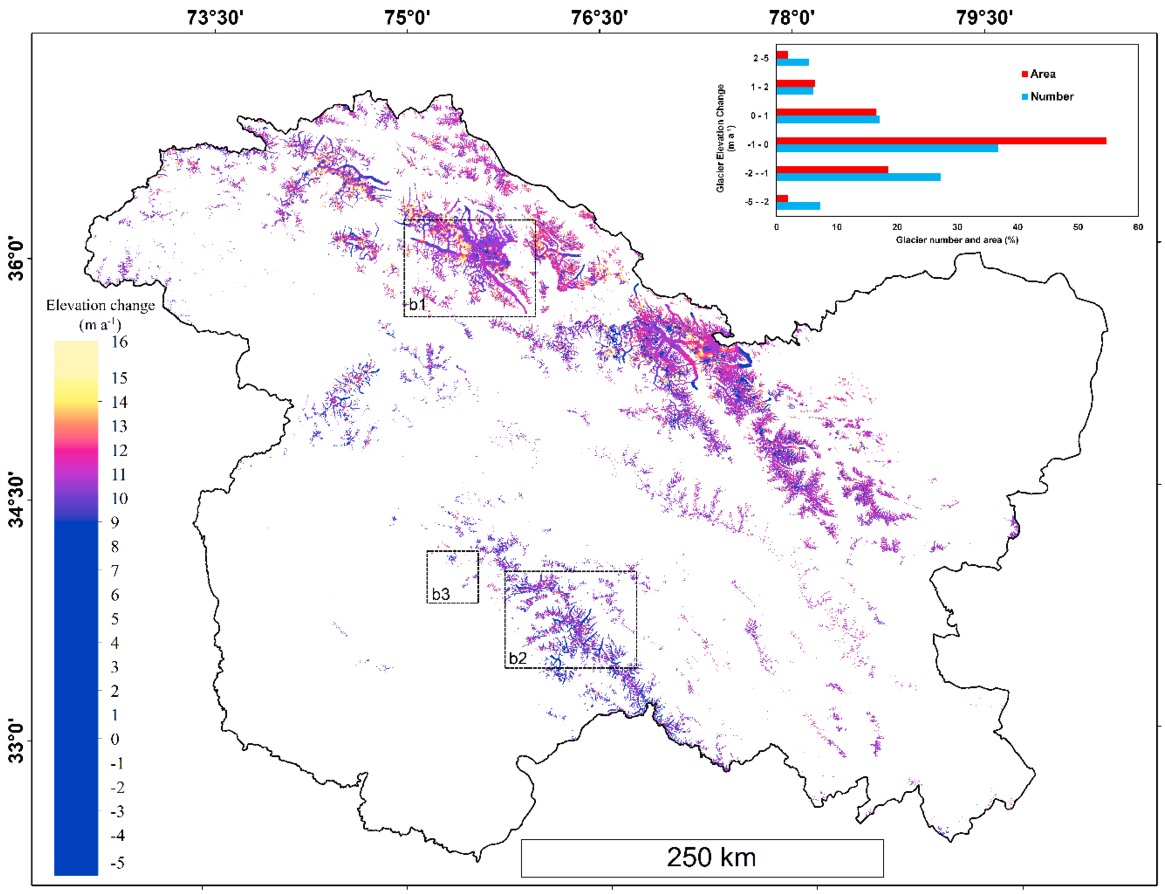

Supplementary **Fig. S3:** Extrapolated glacier thickness changes in UIB. The distribution of glacier thickness change categories in terms of number and area is also indicated by bar graph in the figure. Zoomed in view of the insets b1, b2 and b3 are presented in the Supplementary Fig. S4 and Fig S5. The elevation change categories are based on the mean glacier-wide elevation changes.

~68% of the glaciers (count), accounting for ~73% of the glacier area in the basin, fall in the 0 to -2 thickness change category. 23.30% (count) of the glaciers accounting 23.0 % of the glacier area fall in the thickness change category of 0-2 m a^{-1}. The thickness changes ranging from -2 to -5 m a^{-1} and 2 to 16 m a^{-1} hold only 7.12% of glaciers (equivalent to 1.85% of the total glacier area) and 5.39% (equivalent to 1.95% of the total glacier area) of the glaciers respectively

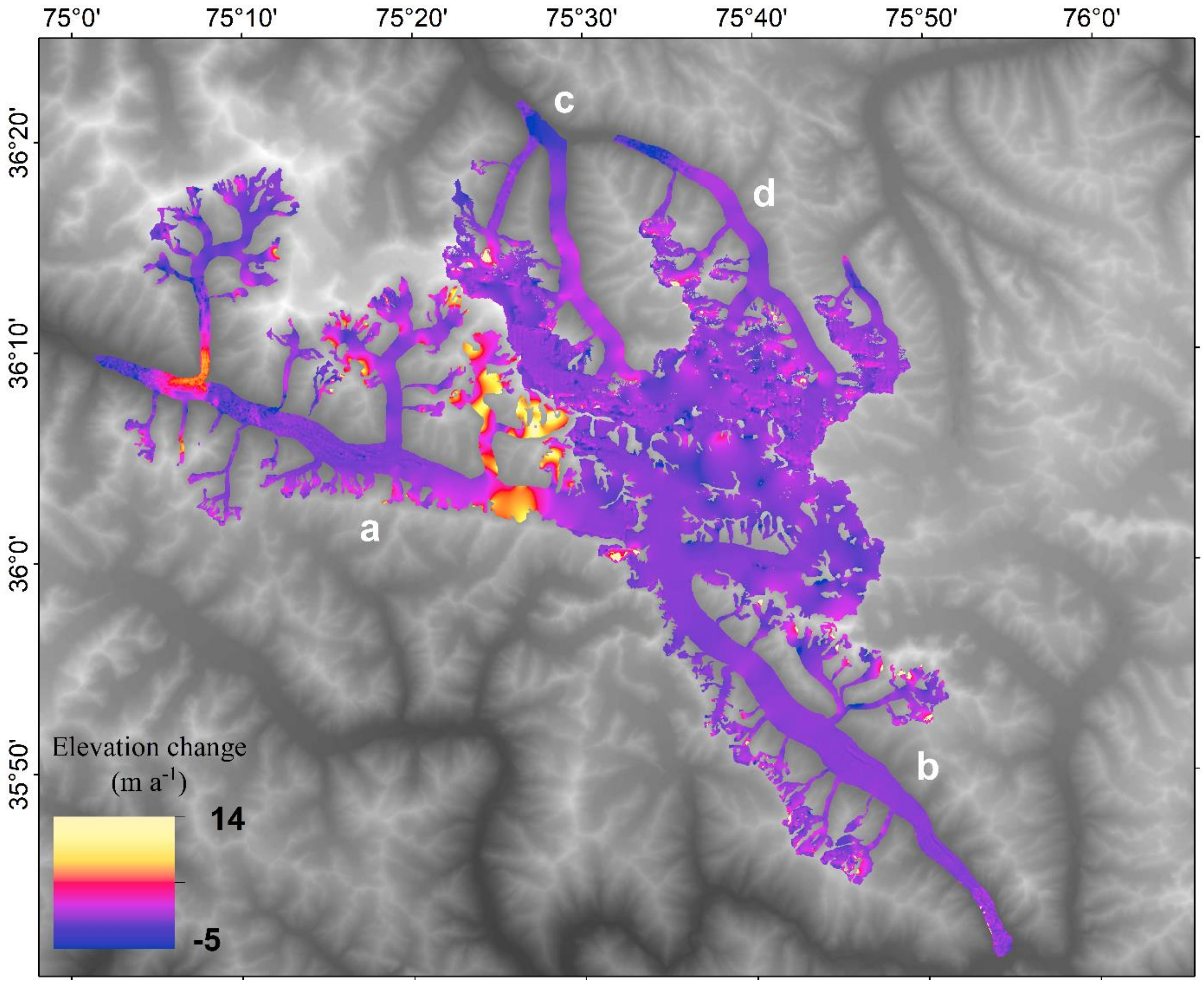

Supplementary **Fig. S4:** Zoomed in view of the inset b1 in Fig S3. The figure presents the extrapolated elevations changes of a) Hispar; b) Biafo; c) Khurdopin and d) Virerab glaciers in the Karakoram mountain range. The elevation gain near the terminus (where the tributary joins the main trunk) and in the lower accumulation zone of the Hispar glacier (near letter a) correlates well with the recent studies that have reported the surging of this glacier[49]. They also reported that the surge does not reach the terminus of the glacier which is also depicted by the considerable thickness loss at the glacier snout indicated in this study.

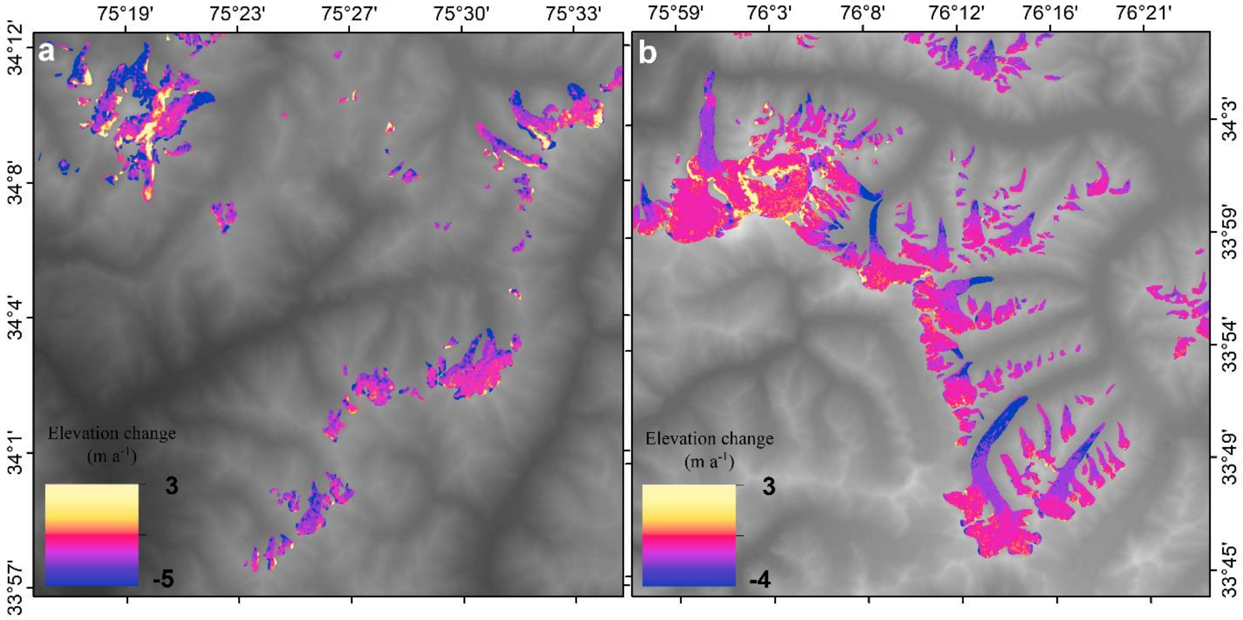

Supplementary **Fig. S5:** Zoomed in view of the insets b2 (right panel of the figure) and b3 (left panel of the figure) of Fig. S3. The figure depicts; a) extrapolated glacier elevations changes in Jhelum basin in the Greater Himalaya range; and b) Suru basin in the Zanaskar mountain range. Higher elevation changes towards the termini of the glaciers are well depicted in the figure.

As indicated by the Fig. S4 and Fig. S5, the elevation gain rates are mostly confined to the higher altitudes (upper parts) of the glaciers and we believe that snow avalanche are responsible for the mass again in these areas.

8. **Details of the data set used** (Supplementary Table S2)

Supplementary **Table S2:** Description of the dataset used in the present study

Dataset	Acquisition date	Source
1. SRTM-C (90 m)	2000	http://srtm.csi.cgiar.org
2. TanDEM-X (90 m)	2011-2015	https://download.geoservice.dlr.de/TDM90/
3. Randolph Glacier Inventory (RGI) 6.0	2000 ± 2	https://www.glims.org/RGI/rgi60_dl.html
4. Supraglacial Debris Cover Dataset v1.0	2000 ± 2	[48]
5. MODIS LST (MOD11A2, 1 km)	2000-2014	https://modis.gsfc.nasa.gov/data/dataprod/mod11.php

69.97% of the glaciers were mapped on satellite images between 2000 and 2002; 28.89% between 1998 and 1999; 1.12% glaciers were mapped on images between 2006 and 2009.

9. Influence of glacier morphological and topographical parameters thickness change

Supplementary **Fig. S6**: Control of glacier elevation on glacier thickness changes. The glacier elevation categories are based on the mean glacier elevation. Area SA is the glacier area distributed on slopes with mean south aspect.

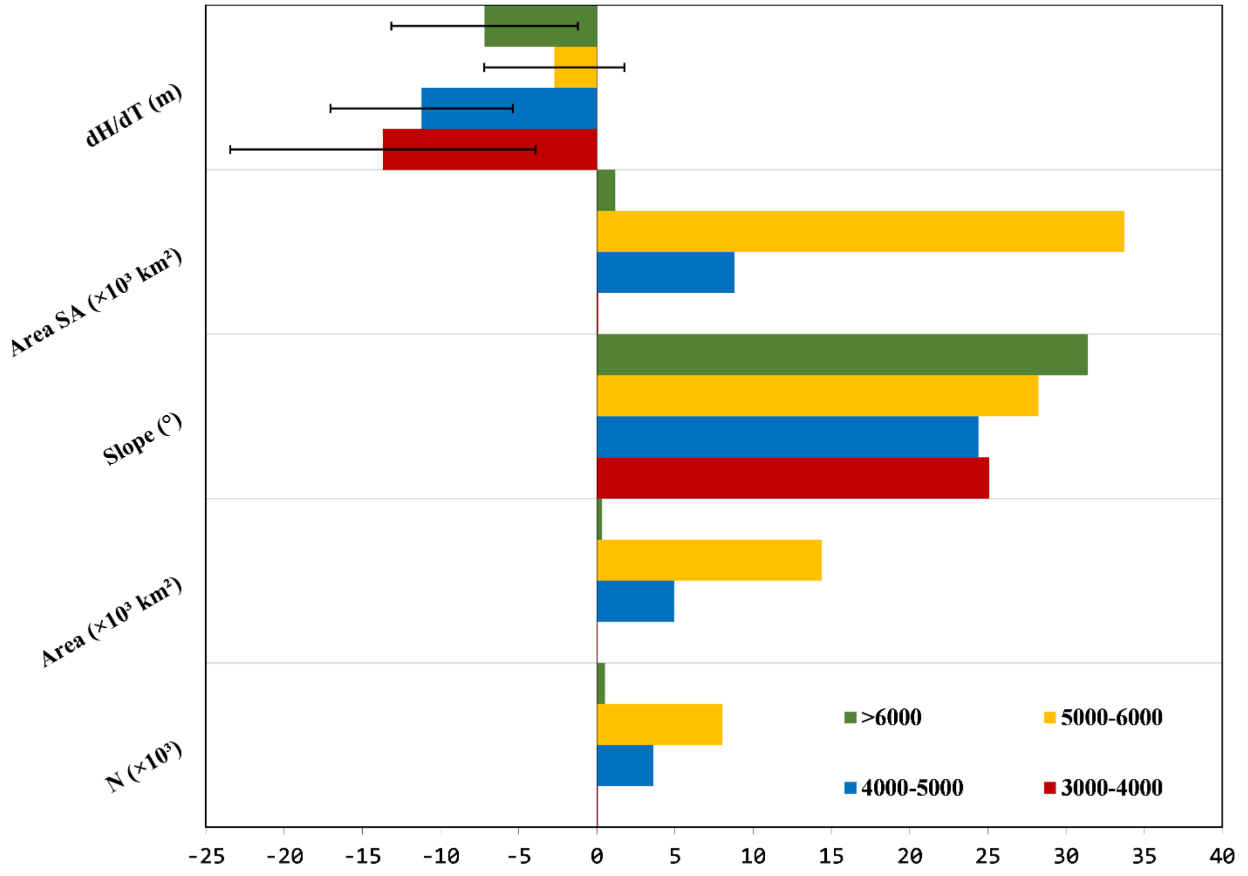

Supplementary **Fig. S7:** Influence of glacier slope on glacier thickness change. The slope categories are based on the mean glacier slope. Area SA is the glacier area distributed on slopes with mean south aspect.

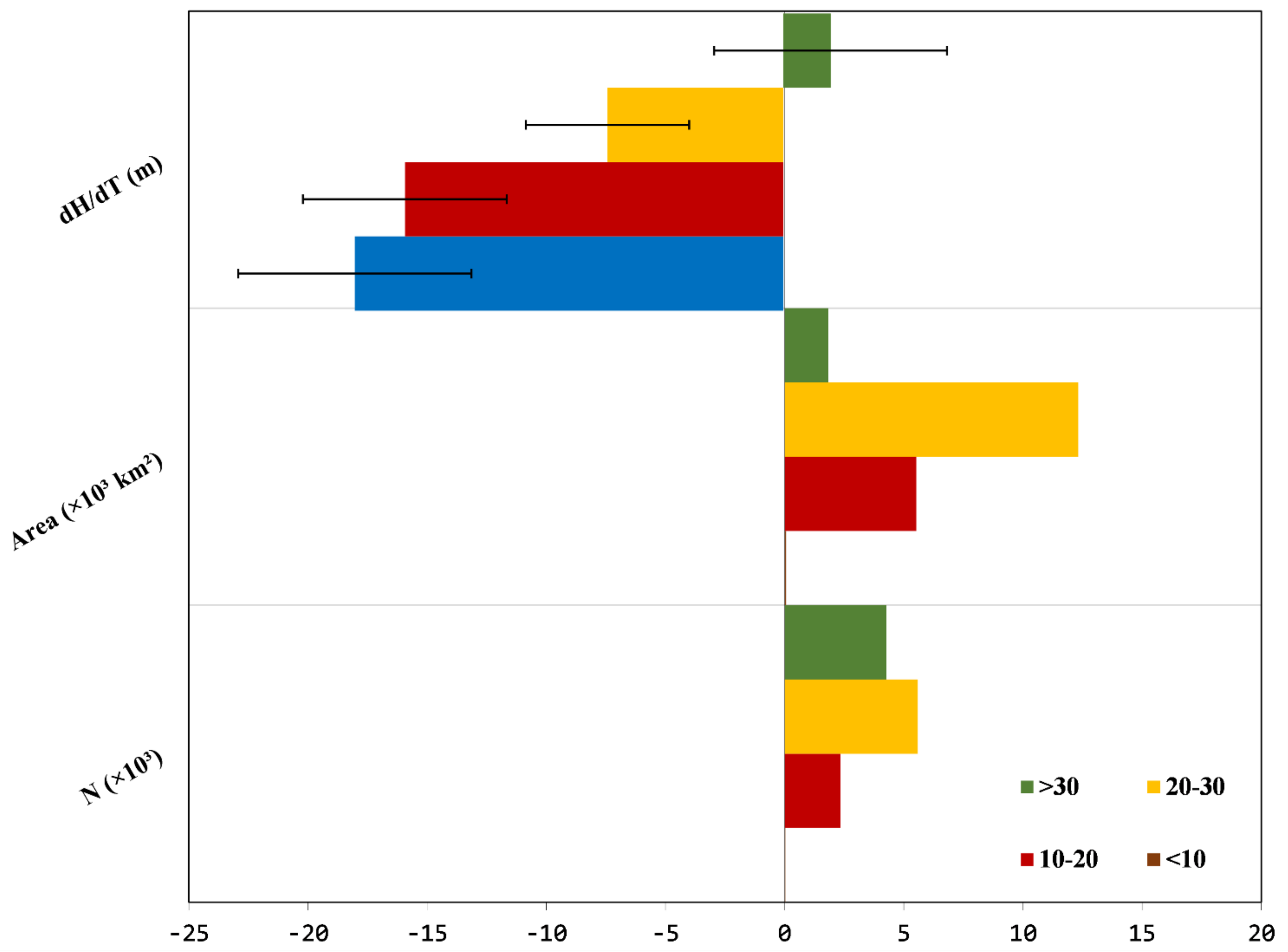

Supplementary **Fig. S8:** Glacier thickness change variability as a function of glacier aspect. The aspect categories are based on the mean glacier aspect. Area SA is the glacier area distributed on slopes with mean south aspect.

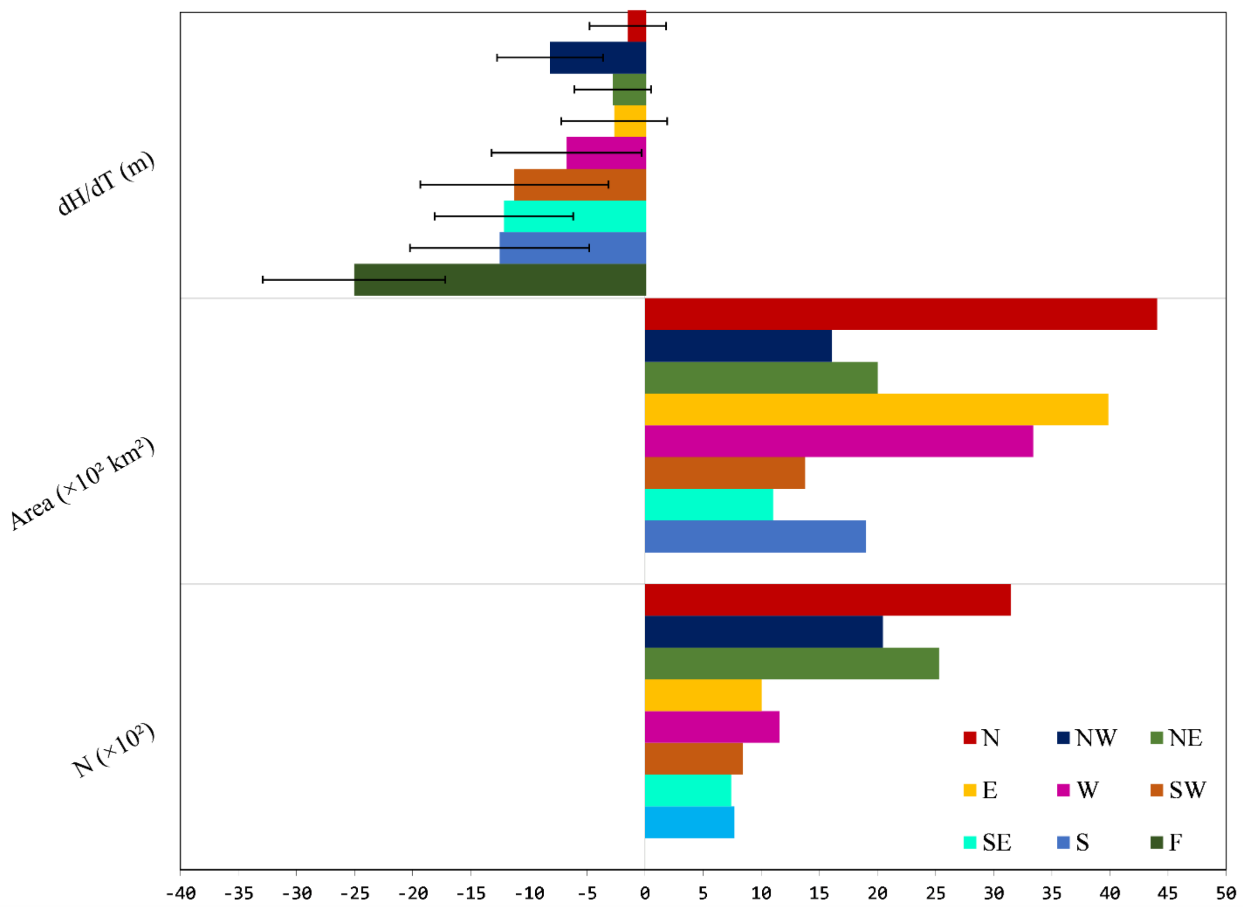

Supplementary **Table S3:** Glacier thickness change variability as a function of glacier altitude across different mountain ranges of the study region.

	**PPR**		
**Elevation (m a.s.l.)**	**Number**	**Area (km^2)**	**dH/dT ($m\ a^{-1}$)**
3000-4000	18	3.45	-2.03 ± 0.67
4000-5000	88	23.03	-1.86 ± 0.59
	**GHR**		
3000-4000	4	0.91	-1.55 ± 0.47
4000-5000	239	106.70	-1.03 ± 0.41
	**SR**		
3000-4000	14	21.27	-1.85 ± 0.58
4000-5000	734	475.76	-1.29 ± 0.44
5000-6000	123	132.84	-0.47 ± 0.30
>6000	7	9.75	-0.46 ± 0.41
	**ZR**		
3000-4000	10	9.17	-0.88 ± 0.35
4000-5000	850	1137.33	-1.36 ± 0.41
5000-6000	853	1206.63	-1.32 ± 0.39
>6000	7	2.34	-2.50 ± 0.67
	**LR**		
4000-5000	90	29.58	-0.71 ± 0.39
5000-6000	3280	2194.15	-0.42 ± 0.25
>6000	347	246.03	-0.70 ± 0.26
	**KKR**		
4000-5000	1633	3198.25	-0.21 ± 0.32
5000-6000	3790	10846.51	-0.23 ± 0.31
>6000	156	84.28	-0.15 ± 0.46
	**UIB**		
3000-4000	46	34.80	-1.40 ± 0.53
4000-5000	3634	4970.65	-0.32 ± 0.39
5000-6000	8046	14380.13	-0.26 ± 0.30
>6000	517	340.05	-0.59 ± 0.37

Supplementary **Table S4:** Glacier thickness change variability as a function of mean glacier aspect across different mountain ranges of the study region.

Slope (°)	Number	Area (km^2)	dH/dT ($m\ a^{-1}$)
		**PPR**	
10-20	50	18.04	-2.04 ± 0.65
20-30	50	7.65	-1.60 ± 0.52
>30	6.00	0.80	-0.39 ± 0.37
		**GHR**	
<10	1	0.56	-2.50 ± 0.75
10-20	85	68.22	-1.21 ± 0.47
20-30	132	36.49	-0.75 ± 0.27
>30	25	2.35	-0.69 ± 0.44
		**SR**	
<10	3	1.17	-2.19 ± 0.62
10-20	355	340.78	-1.39 ± 0.47
20-30	418	262.27	-0.88 ± 0.41
>30	102	35.40	-0.37 ± 0.49
		**ZR**	
<10	10	48.25	-1.58 ± 0.51
10-20	637	1710.50	-1.43 ± 0.41
20-30	749	494.77	-1.14 ± 0.38
>30	324	101.94	-0.81 ± 0.42
		**LR**	
<10	14	8.79	-1.03 ± 0.29
10-20	825	1021.76	-0.76 ± 0.29
20-30	1917	1159.13	-0.32 ± 0.22
>30	961	280.08	0.02 ± 0.33
		**KKR**	
<10	4	0.24	-0.90 ± 0.33
10-20	400	2360.27	-0.54 ± 0.26
20-30	2321	10347.03	-0.04 ± 0.25
>30	2854	1421.49	0.49 ± 0.39
		**UIB**	
<10	32	59.01	-1.52 ± 0.40
10-20	2352	5519.56	-0.95 ± 0.35
20-30	5587	12307.34	-0.14 ± 0.28
>30	4274	1842.06	0.28 ± 0.40

Supplementary **Table S5:** Glacier thickness change variability as a function of the mean glacier aspect across different mountain ranges of the study region.

	**PPR**		
**Aspect**	**Number**	**Area (km^2)**	**dH/dT (m a^{-1})**
S	1	0.11	-2.33 ± 0.70
SE	6	1.24	-2.55 ± 0.72
SW	5	0.91	-2.76 ± 0.87
W	8	1.23	-2.53 ± 0.78
E	6	0.75	-1.45 ± 0.58
NE	27	7.35	-1.67 ± 0.50
NW	18	3.82	-1.99 ± 0.55
N	35	11.07	-1.45 ± 0.52
	**GHR**		
S	11	5.65	-1.27 ± 0.53
SE	5	2.64	-2.29 ± 0.67
SW	12	7.41	-1.00 ± 0.58
W	20	11.94	-1.40 ± 0.46
E	13	3.75	-1.28 ± 0.37
NE	88	44.62	-0.05 ± 0.38
NW	50	14.39	-1.17 ± 0.43
N	44	17.21	-0.80 ± 0.21
	**SR**		
F	2	0.21	-1.91 ± 0.59
S	48	37.84	-1.22 ± 0.63
SE	57	52.54	-1.71 ± 0.56
SW	69	32.33	-1.59 ± 0.54
W	120	63.88	-1.37 ± 0.43
E	81	41.00	-1.36 ± 0.39
NE	161	134.24	-1.28 ± 0.32
NW	153	151.23	-0.66 ± 0.34
N	187	126.34	-0.93 ± 0.33
	**ZR**		
S	160	163.70	-1.77 ± 0.55
SE	148	177.52	-1.57 ± 0.51
SW	158	280.85	-1.71 ± 0.54
W	125	163.40	-1.53 ± 0.43
E	190	219.53	-1.44 ± 0.39
NE	278	390.36	-1.21 ± 0.32
NW	263	335.80	-1.11 ± 0.34
N	398	624.30	-0.65 ± 0.33

**LR**			
S	81	53.35	-1.12 ± 0.46
SE	110	90.66	-1.04 ± 0.43
SW	59	38.15	-1.03 ± 0.48
W	152	85.40	-0.78 ± 0.30
E	272	247.32	-0.65 ± 0.28
NE	992	707.47	-0.38 ± 0.23
NW	716	307.27	-0.42 ± 0.25
N	1335	940.15	-0.33 ± 0.26
**KKR**			
S	469	1640.51	-0.21 ± 0.41
SE	415	777.76	-0.34 ± 0.41
SW	542	1019.73	-0.31 ± 0.35
W	581	1288.46	-0.19 ± 0.36
E	590	2894.74	-0.37 ± 0.30
NE	1023	2661.24	-0.01 ± 0.27
NW	855	1189.26	0.22 ± 0.31
N	1104	2657.33	0.15 ± 0.33
**UIB**			
F	2	0.211	-1.91 ± 0.59
S	770	1901.17	-0.38 ± 0.23
SE	741	1102.37	-0.67 ± 0.43
SW	844	1378.53	-0.60 ± 0.47
W	1158	3340.85	-0.14 ± 0.40
E	1004	3986.89	-0.47 ± 0.33
NE	2529	2002.25	-0.25 ± 0.27
NW	2047	1607.74	-0.23 ± 0.30
N	3148	4406.98	-0.20 ± 0.20

Supplementary **Table S6:** Glacier thickness change: Clean vs debris-covered glaciers

Glacier Status	dH/dT (m a^{-1})	
	Criterion 1	Criterion 2
Clean	-0.35 ± 0.33 (11232*)	-0.29 ± 0.33 (11633)
Sparsely debris-covered	-	-0.78 ± 0.36 (502)
Debris-covered	-0.53 ± 0.35 (1011)	-0.47 ± 0.41 (108)

*number of glaciers in each category

Supplementary **Table S7:** Glacier thickness change as a function of glacier size

Size	Number	Area		dH/dT	Mean Elevation	Slope	Area (south aspect)		Debris cover	
km^2	N	km^2	%	m a^{-1}	m	°	km^2	%	km^2	%
<1	9916	2574.94	13.05	-0.43 ± 0.35	5292	28.21	482.24	18.72	152.73	5.93
1-2	1023	1443.32	7.32	-0.48 ± 0.26	5279	23.57	276.42	19.15	86.80	6.01
2-5	769	2394.23	12.14	-0.46 ± 0.25	5316	22.92	373.31	15.59	157.05	6.55
5-10	268	1838.78	9.32	-0.39 ± 0.23	5322	22.18	419.38	22.80	158.85	8.63
10-20	137	1831.03	9.28	-0.37 ± 0.25	5288	21.95	427.96	23.37	169.80	9.27
20-30	42	1056.31	5.35	-0.59 ± 0.26	5356	20.66	144.71	13.69	102.22	9.67
30-40	23	711.43	3.61	-0.34 ± 0.22	5281	22.43	147.31	20.70	78.50	11.03
40-50	18	714.53	3.62	-0.58 ± 0.34	5101	20.32	171.70	24.03	120.38	16.84
>50	47	7162.65	36.31	-0.06 ± 0.19	5298	22.49	1774.53	24.77	797.91	11.13

Supplementary **Table S8:** Mean winter, summer and annual temperatures over different mountain ranges in the study region derived from MODIS LST

	Temperature (°C)		
**Mountain Range**	**Winter**	**Summer**	**Annual**
PPR	-4.35	16.45	6.05
GHR	-5.49	14.52	4.51
SR	-6.10	16.32	5.38
ZR	-11.12	12.72	0.80
LR	-11.55	12.83	0.64
KKR	-15.49	1.35	-7.06

The temperature values obtained, from MODIS 8-day LST MOD11A2 product, are the mean temperature values for each month and each mountain range. The mean monthly values were then averaged for the winter (Nov-April) and summer (May-Oct.) time periods. The winter and summer temperatures presented in the manuscript are averaged over a period of 14 years (2000-2014).

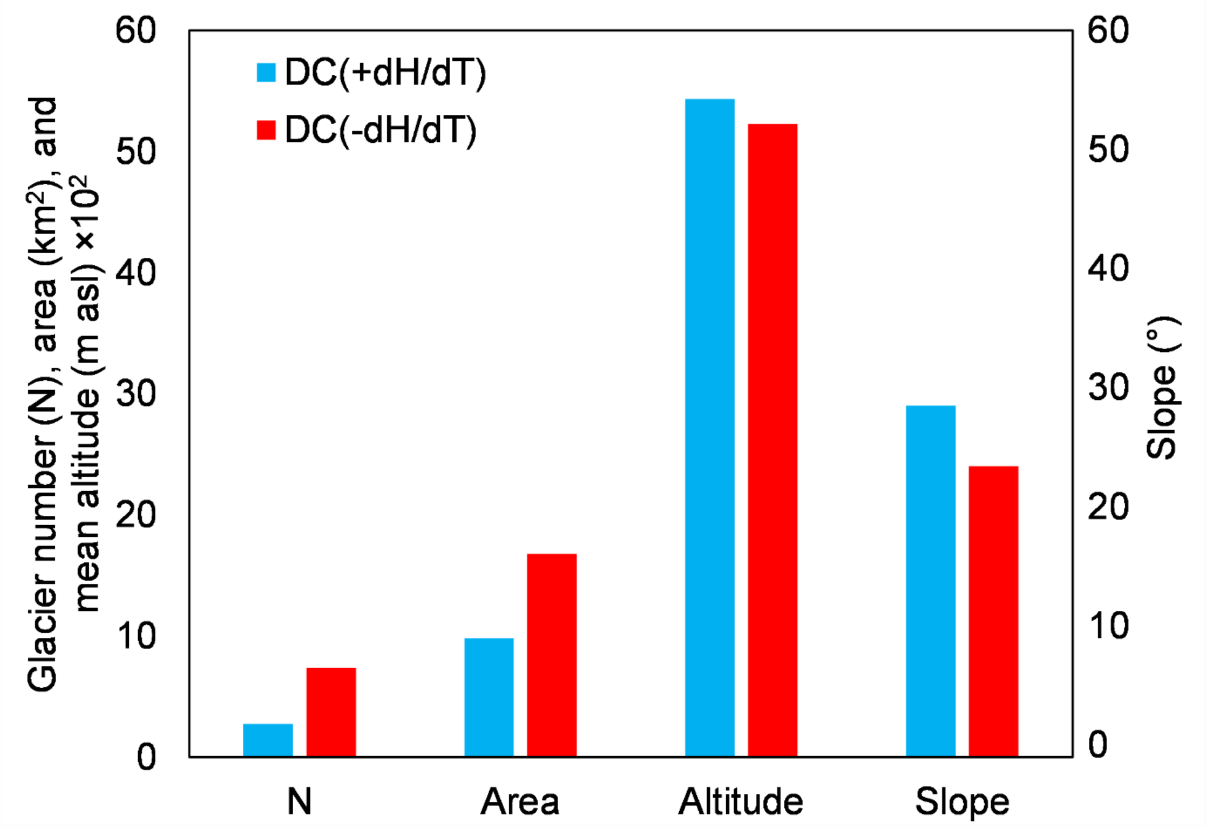

Supplementary **Fig. S9:** Morphology of debris-covered glaciers with positive and negative thickness changes. The glacier classification is based on Criterion 1. The figure indicates no uniform thickness change in the debris-covered glaciers and the variability in the thickness change is elucidated by topographic parameters mean elevation and mean slope. Debris-covered glaciers with positive thickness or no thickness change are located at higher altitudes and have relatively higher slopes compared to the debris-covered glaciers with negative thickness changes.

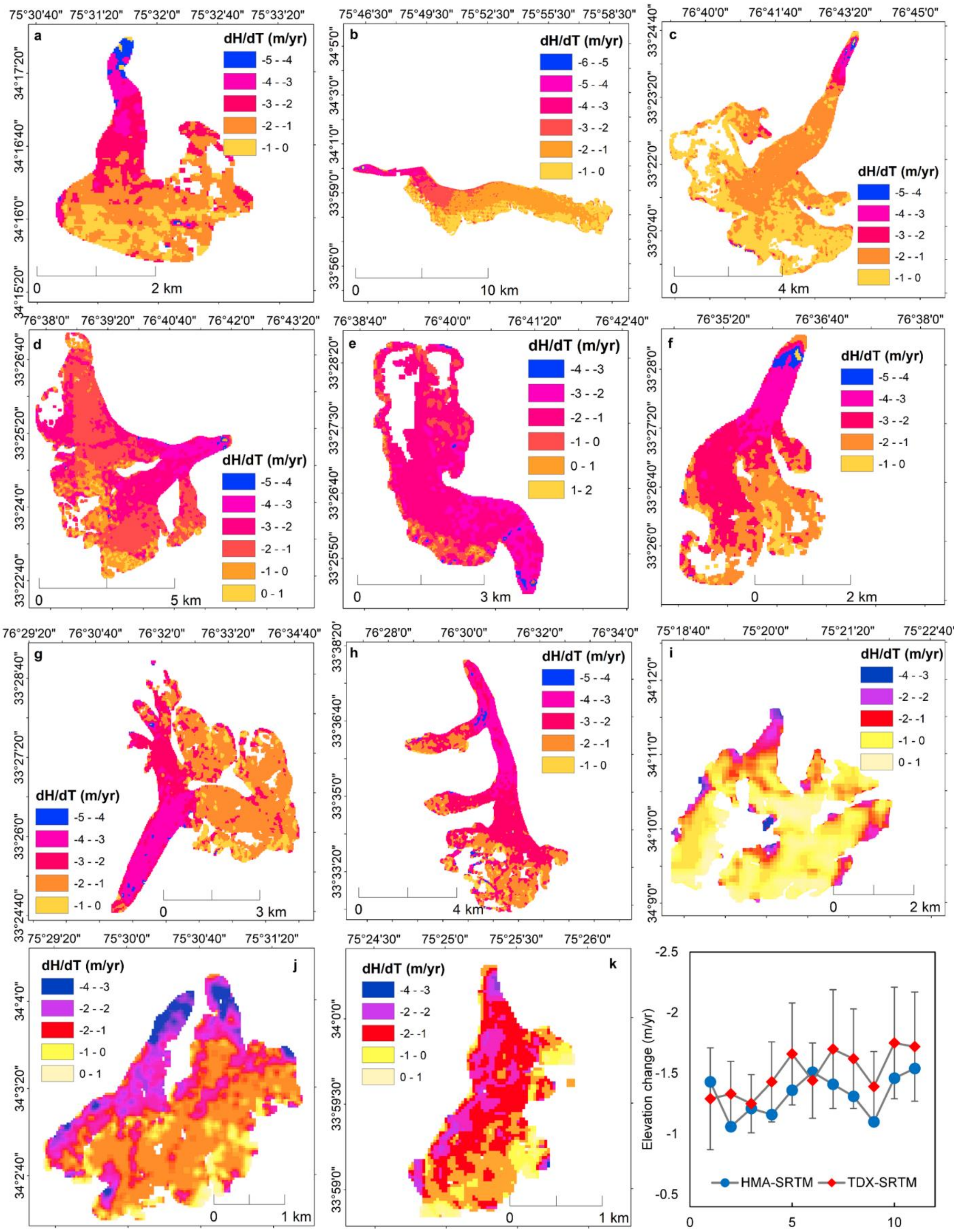

Supplementary **Fig. S10:** Glacier elevation changes of the selected 11 glaciers in the study area based on the HMA and SRTM DEM. a) Machoi Glacier (GLIMS ID: G076665E33451N); b)

Brankton Glacier (G075881E33976N); c) G076691E33361N; d) G076642E33428N; e) G076665E33451N; f) G076594E33444N; g) G076561E33435N; h) G076513E33579N; i) Kolahoi Glacier (G075315E34163N); j) Shishram Glacier (G075511E34051N) ; k) G075422E33990N. The comparison of elevation changes based on TanDEM (TDX)-SRTM and HMA-SRTM is provided in the last panel of the figure. The details of HMA DEMs used is given below:

1. HMA_DEM8m_CT_20090823_0554_1020010009762500_10200100088A38001.tif
2. HMA_DEM8m_CT_20120709_1753_102001001C00F200_103001001A5D91001.tif
3. HMA_DEM8m_CT_20130127_0543_10504100010D8200_102001001FAD23001.tif
4. HMA_DEM8m_CT_20130211_0542_1020010021D94400_102001002013BF001.tif
5. HMA_DEM8m_CT_20130521_0547_1030010023770B00_10200100223F51001.tif
6. HMA_DEM8m_CT_20131026_0546_10200100269B2B00_102001002789D5001.tif
7. HMA_DEM8m_CT_20131026_0547_102001002466BA00_102001002532B3001.tif
8. HMA_DEM8m_CT_20131026_0547_10200100269B2B00_102001002532B3001.tif
9. HMA_DEM8m_CT_20141025_1736_1040010003AC5200_10300100388CC8001.tif
10. HMA_DEM8m_CT_20141129_1801_103001003CC3F400_1020010038C4A9001.tif
11. HMA_DEM8m_CT_20150506_1819_1030010043C6F800_102001003E585D001.tif
12. HMA_DEM8m_CT_20130211_0542_1020010021D94400_102001001F7A18001.tif

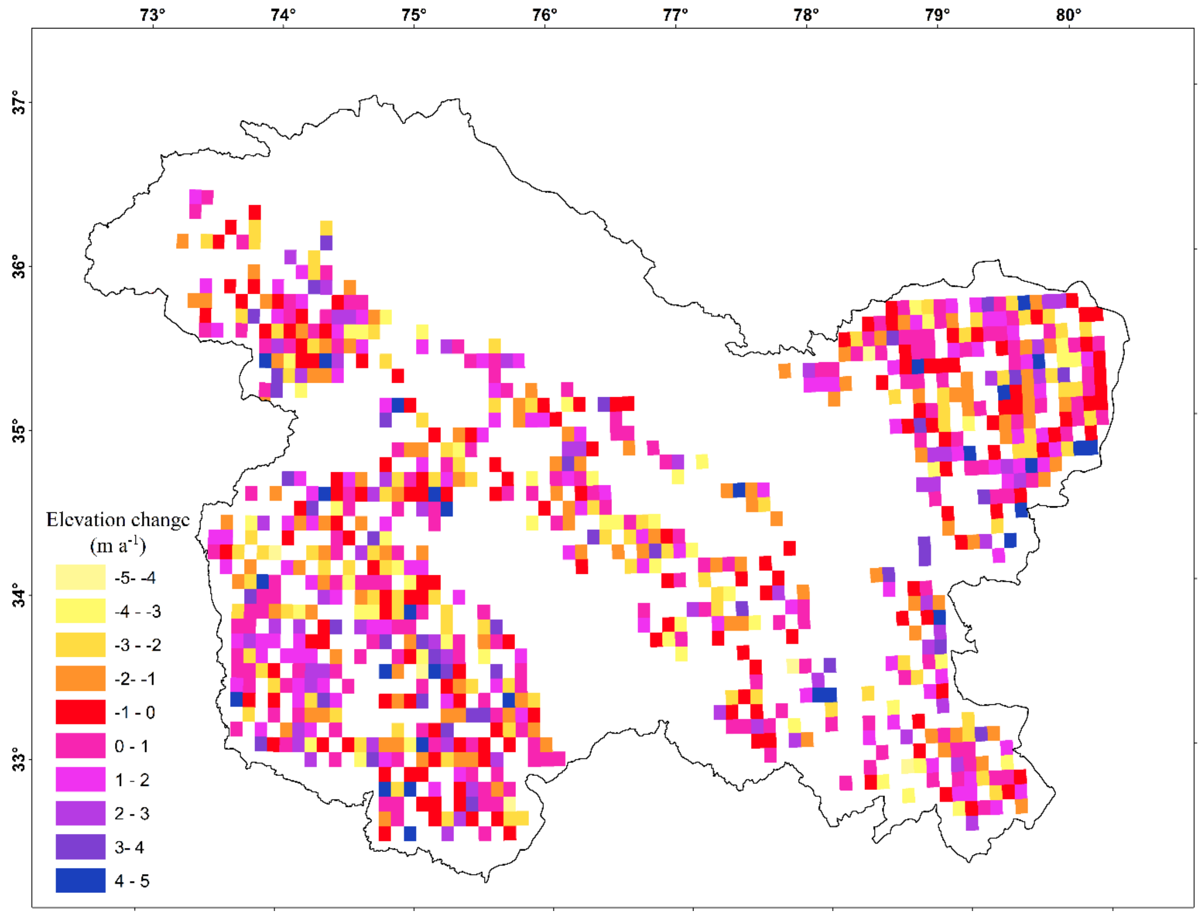

Supplementary **Fig. S11:** Off-glacier elevation changes at 10×10 km grid for the study region. Mean off-glacier elevation change of -0.06 m was observed for the study region

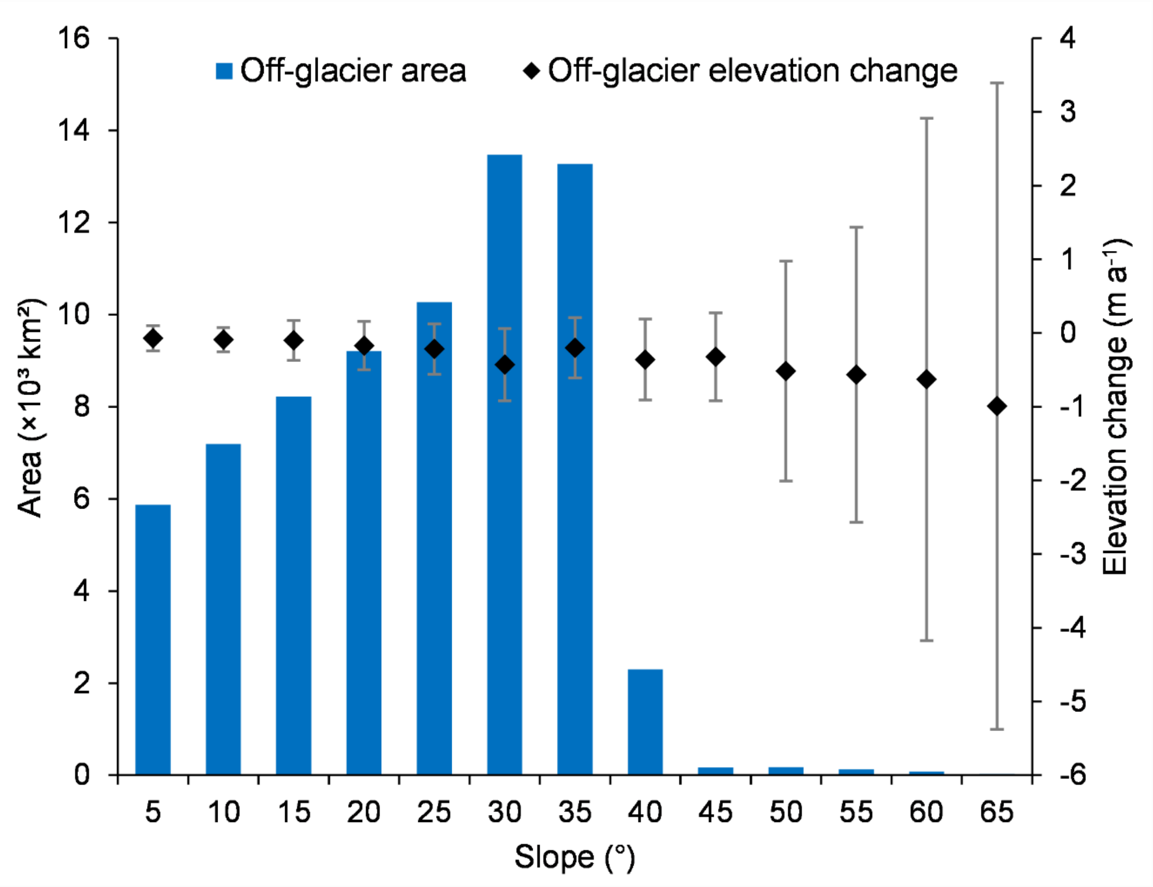

Supplementary Fig. S12: Off-glacier area (blue bars) and off-glacier elevation change (black diamonds) derived at 5° slope bins for the study region. The error bars indicate NMAD in each slope range.

References

1. Van Niel, T. G., McVicar, T. R., Li, L., Gallant, J. C. & Yang, Q. The impact of misregistration on SRTM and DEM image differences. *Remote Sensing of Environment*, **112**, 2430-2442, (2008).
2. Nuth, C. & Kääb, A. Co-registration and bias corrections of satellite elevation data sets for quantifying glacier thickness change. *The Cryosphere*, **5**, 271-290, (2011).
3. Purinton, B. & Bookhagen, B. Measuring decadal vertical land-level changes from SRTM-C (2000) and TanDEM-X (∼ 2015) in the south-central Andes. *Earth Surface Dynamics*, **6**, 971-987, (2018).
4. Le Bris, R., & Paul, F. Glacier-specific elevation changes in parts of western Alaska. *Annals of Glaciology*, **56**, 184-192, (2015).
5. Brun, F., Berthier, E., Wagnon, P., Kääb, A., & Treichler, D. A spatially resolved estimate of High Mountain Asia glacier mass balances from 2000 to 2016. *Nature Geoscience*, **10**, 668-673, (2017).
6. Huber, J., McNabb, R., & Zemp, M. Elevation changes of west-central Greenland glaciers from 1985 to 2012 from remote sensing. *Frontiers in Earth Science*, **8**, 35, (2020).
7. McNabb, R. W., Nuth, C., and Kääb, A. Phase 2: option 2, algorithm development: Voids. Technical Report. Glaciers_cci-D1.2_LAR, European Space Agency Glaciers CCI Project, (2017).
8. McNabb, R., Nuth, C., Kääb, A., and Girod, L. Sensitivity of glacier volume change estimation to DEM void interpolation. *Cryosphere*, **13**, 895-910, (2019).
9. Watson, D. F. Contouring: A Guide to the Analysis and Display of Spatial Data, Pergamon, Oxford, (1992).
10. Kalkhan, M. A. Spatial statistics: geospatial information modeling and thematic mapping. (CRC Press, 2011).
11. RGI Consortium. Randolph Glacier Inventory (RGI) – A Dataset of Global Glacier Outlines: Version 6.0. Technical Report, Global Land Ice Measurements from Space, Boulder, Colorado, USA. (Digital Media, 2017).
12. Yamazaki, D. *et al*. A high-accuracy map of global terrain elevations. *Geophysical Research Letters*, **44**, 5844-5853, (2017).

13. Purinton, B. & Bookhagen, B. Validation of digital elevation models (DEMs) and comparison of geomorphic metrics on the southern Central Andean Plateau. *Earth Surface Dynamics*, **5**, 211-237, (2017).
14. Baade, J. & Schmullius, C. TanDEM-X IDEM precision and accuracy assessment based on a large assembly of differential GNSS measurements in Kruger National Park, South Africa. *ISPRS Journal of Photogrammetry and Remote Sensing*, **119**, 496-508, (2016).
15. Wessel, B., Huber, M., Wohlfart, C., Marschalk, U., Kosmann, D. & Roth, A. Accuracy assessment of the global TanDEM-X Digital Elevation Model with GPS data. *ISPRS Journal of Photogrammetry and Remote Sensing*, **139**, 171-182, (2018).
16. Carabajal, C. C. & Harding, D. J. SRTM C-band and ICESat laser altimetry elevation comparisons as a function of tree cover and relief. *Photogrammetric Engineering & Remote Sensing*, **72**, 287-298, (2006).
17. Gorokhovich, Y. & Voustianiouk, A. Accuracy assessment of the processed SRTM-based elevation data by CGIAR using field data from USA and Thailand and its relation to the terrain characteristics. *Remote Sensing of Environment*, **104**, 409-415, (2006).
18. Gallant, J. C. & Read, A. Enhancing the SRTM data for Australia. *Proceedings of Geomorphometry*, **31**, 149-154, (2009).
19. Rolstad, C., Haug, T. & Denby, B. Spatially integrated geodetic glacier mass balance and its uncertainty based on geostatistical analysis: application to the western Svartisen ice cap, Norway. *Journal of Glaciology*, **55**, 666-680, (2009).
20. Fischer, M., Huss, M. & Hoelzle, M. Surface elevation and mass changes of all Swiss glaciers 1980–2010. *The Cryosphere*, **9**, 525-540, (2015).
21. Gardelle, J., Berthier, E., & Arnaud, Y. Slight mass gain of Karakoram glaciers in the early twenty-first century. *Nature Geoscience*, **5**, 322-325, (2012).
22. Vijay, S., & Braun, M. Early 21st century spatially detailed elevation changes of Jammu and Kashmir glaciers (Karakoram–Himalaya). *Global and Planetary Change*, **165**, 137-146, (2018).
23. Vijay, S., & Braun, M. (2016). Elevation change rates of glaciers in the Lahaul-Spiti (Western Himalaya, India) during 2000–2012 and 2012–2013. *Remote Sensing*, **8**, 1038.
24. Huber, J., McNabb, R., & Zemp, M. Elevation changes of west-central Greenland glaciers from 1985 to 2012 from remote sensing. *Frontiers in Earth Science*, **8**, 35, (2020).

25. Brun, F. *et al*. Heterogeneous influence of glacier morphology on the mass balance variability in High Mountain Asia. *Journal of Geophysical Research: Earth Surface*, **124**, 1331-1345, (2019).
26. Seehaus, T. *et al*. Changes of the tropical glaciers throughout Peru between 2000 and 2016–mass balance and area fluctuations. *The Cryosphere*, **13**, 2537-2556, (2019).
27. Höhle, J. & Höhle, M. Accuracy assessment of digital elevation models by means of robust statistical methods. *ISPRS Journal of Photogrammetry and Remote Sensing*, **64**, 398-406, (2009).
28. Dormann, F. C. *et al.* Methods to account for spatial autocorrelation in the analysis of species distributional data: a review. *Ecography*, **30**, 609-628, (2007).
29. Braun, A. M. H., Malz, P., Sommer, C. & Barahona, D. F. Constraining glacier elevation and mass changes in South America. *Nature Climate Change Letters*, **9**, 130-136, (2019).
30. Paul, *et al*. On the accuracy of glacier outlines derived from remote-sensing data. *Annals of Glaciology*, **54**, 171-182, (2013).
31. Rastner *et al.* The first complete inventory of the local glaciers and ice caps on Greenland. *The Cryosphere*, **6**, 1483-1495, (2012).
32. Azam *et al.* Review of the status and mass changes of Himalayan-Karakoram glaciers. *Journal of Glaciology*, **64**, 61-74, (2018).
33. Shean, D. E., Joughin, I. R., Dutrieux, P., Smith, B. E., & Berthier, E. Ice shelf basal melt rates from a high-resolution digital elevation model (DEM) record for Pine Island Glacier, Antarctica. *The Cryosphere*, **13**, 2633-2656, (2019).
34. Huss, M. Density assumptions for converting geodetic glacier volume change to mass change. *The Cryosphere*, **7**, 877-887, (2013).
35. Ali, I., Shukla, A. & Romshoo, S. A. Assessing linkages between spatial facies changes and dimensional variations of glaciers in the upper Indus Basin, western Himalaya. *Geomorphology*, **284**, 115-129, (2017).
36. Mattson, L. E. Ablation on debris covered glaciers: an example from the Rakhiot Glacier, Punjab, Himalaya. *International Association of Hydrological Sciences*, **218**, 289-296, (1993).
37. Nakawo, M. Satellite data utilization for estimating ablation of debris covered glaciers. *International Association of Hydrological Sciences*, **218**, 75-83, (1993).

38. Xiang, Y. *et al*. Retreat rates of debris-covered and debris-free glaciers in the Koshi River Basin, central Himalayas, from 1975 to 2010. *Environmental Earth Sciences*, **77**, 285, (2018).
39. Janke, J. R., Bellisario, A. C. & Ferrando, F. A. Classification of debris-covered glaciers and rock glaciers in the Andes of central Chile. *Geomorphology*, **241**, 98-121, (2015).
40. Shekhar, M. S., Devi, U., Dash, S.K., Singh, G. P. & Singh, A. Variability of Diurnal Temperature Range During Winter Over Western Himalaya: Range-and Altitude-Wise Study. *Pure and Applied Geophysics*, **175**, 3097-3109, (2018).
41. Nuimura, T. *et al.* The GAMDAM glacier inventory: a quality-controlled inventory of Asian glaciers, *The Cryosphere*, **9**, 849–864, (2015).
42. Frey, H., Paul, F., & Strozzi, T. Compilation of a glacier inventory for the western *Himalayas from satellite data: Methods, challenges, and results. Remote Sensing of Environment*, **124**, 832–843, (2012).
43. Bajracharya, S. R. & Shrestha, B. The status of glaciers in the Hindu Kush-Himalayan region. Kathmandu: ICIMOD, (2011).
44. ISRO. Snow and glaciers of Himalayas: inventory and monitoring. Space Applications Centre, ISRO, India, (2010).
45. Raina, V. K. Himalayan glaciers: a state-of-art review of glacial studies, glacial retreat and climate change. p 60 (discussion paper, Ministry of Environment and Forests, Government of India, New Delhi), (2009).
46. Wester P., Mishra A., Mukherji A. & Shrestha A. B. The HinduKush Himalaya Assessment—Mountains, Climate Change, Sustainability and People. Springer Nature Switzerland AG, Cham, (2019).
47. Scherler, D., Bookhagen, B. & Strecker, M.R. Spatially variable response of Himalayan glaciers to climate change affected by debris cover. *Nature Geoscience*, **4**, 156-159, (2011).
48. Scherler, D., Wulf, H. & Gorelick, N. Global Assessment of Supraglacial Debris-Cover Extents. *Geophysical Research Letters*, **45**, 11-798, (2018).
49. Rashid, I., Abdullah, T., Glasser, N. F., Naz, H., & Romshoo, S. A. Surge of Hispar Glacier, Pakistan, between 2013 and 2017 detected from remote sensing observations. *Geomorphology*, **303**, 410-416, (2018).
